# Prenatal exposure to benzodiazepine and z-hypnotics and fifth-grade scholastic skills—emulating target trials using data from the Norwegian Mother, Father and Child Cohort Study

**DOI:** 10.1093/aje/kwae159

**Published:** 2024-06-29

**Authors:** Lene Maria Sundbakk, Mollie Wood, Jon Michael Gran, Hedvig Nordeng

**Affiliations:** PharmacoEpidemiology and Drug Safety Research Group, Department of Pharmacy, Faculty of Mathematics and Natural Sciences, University of Oslo, 0316 Oslo, Norway; PharmaTox Strategic Initiative, Faculty of Mathematics and Natural Sciences, University of Oslo, 0316 Oslo, Norway; Department of Epidemiology, Gillings School of Global Public Health, University of North Carolina at Chapel Hill, Chapel Hill, North Carolina 27599, United States; Oslo Centre for Biostatistics and Epidemiology, Department of Biostatistics, University of Oslo, 0317 Oslo, Norway; PharmacoEpidemiology and Drug Safety Research Group, Department of Pharmacy, Faculty of Mathematics and Natural Sciences, University of Oslo, 0316 Oslo, Norway; PharmaTox Strategic Initiative, Faculty of Mathematics and Natural Sciences, University of Oslo, 0316 Oslo, Norway; Department of Child Health, Norwegian Institute of Public Health, 0473 Oslo, Norway

**Keywords:** prenatal exposure, pharmacoepidemiology, pregnancy, mental health, target trial emulation, child neurodevelopment, school performance, Norwegian Mother, Father and Child Cohort Study (MoBa)

## Abstract

Evidence is limited regarding the effect of prenatal benzodiazepine and z-hypnotic exposure and long-term neurodevelopment in childhood. The objective of this study was to investigate the effects of initiating benzodiazepine or z-hypnotic treatment in early, mid, and late pregnancy on fifth-grade numeracy and literacy scholastic skills in children by emulating 3 target trials. The trials are identical except for the timing of enrollment and the number of eligible individuals. Eligibility to the trials required a history of anxiety and/or depression prior to pregnancy. We used data from the Norwegian Mother, Father and Child Cohort Study, linked to the Medical Birth Registry of Norway, to emulate the trials. We adjusted for baseline covariates that were available at time 0 for each trial by inverse probability of treatment weighting using the propensity score. The findings of this study did not show any effect of mothers’ initiation of treatment with benzodiazepines or z-hypnotics in early, mid, or late pregnancy on the children’s fifth-grade test scores in numeracy and literacy. The study results provide reassurance for patients in need of benzodiazepines and z-hypnotics during pregnancy; however, these findings need to be interpreted with caution due to low study power in some of the analyses.

This article is part of a Special Collection on Pharmacoepidemiology.

## Introduction

Benzodiazepines and z-hypnotics are drugs mainly prescribed for the treatment of anxiety and insomnia.^1^ Up to 15% experience anxiety disorders during pregnancy, often comorbid with depression.^2^^,^^3^ The global prevalence of prescribed benzodiazepines and z-hypnotics during pregnancy is estimated at 1.9%.^4^ Maternal benzodiazepine or z-hypnotic use may have the potential to influence fetal neurodevelopment,^5^^,^^6^ but studies on child neurodevelopment after prenatal exposure to benzodiazepines and/or z-hypnotics on child neurodevelopment are limited.^7^^‑^^9^ Scholastic achievements and cognitive abilities are important for children’s daily lives and may influence future outcomes such as academic success, income, and socioeconomic status^10^^‑^^12^; however, these outcomes are rarely assessed in perinatal pharmacoepidemiologic studies.^13^^‑^^16^

Perinatal pharmacoepidemiologic studies using observational data are vulnerable to time-related biases,^17^ including immortal time bias.^18^^,^^19^ The target trial emulation framework is one approach to addressing time-related bias.^20^^,^^21^ This approach emphasizes the alignment of eligibility to the trial, treatment assignment, and start of follow-up at time zero to reduce potential immortal time bias.^22^ The target trial emulation framework was recently extended to the pregnancy setting,^23^ with the suggestion that target trials may need to be specific to gestational age, as vulnerability to exposures varies over the course of pregnancy, as does the potential for selection bias.

Benzodiazepines and z-hypnotics are used intermittently,^24^ and their indication for use (anxiety or insomnia) also changes over time. The etiologically relevant window during pregnancy for exposure is unknown, and it is possible that exposure in some parts of pregnancy is safer than others.^25^^,^^26^ By using the target trial emulation framework, we emulated trials for benzodiazepine and z-hypnotic initiation in 3 time periods in pregnancy. The aim of this study was to estimate the effects of time-specific exposures to benzodiazepines and z-hypnotics on fifth-grade literacy and numeracy test scores.

## Methods

### Target trials

We outline the protocol of 3 target trials to evaluate the safety of benzodiazepine or z-hypnotic exposure during pregnancy. The trials, summarized in Table 1, are identical except for the timing of enrollment, as proposed by Hernández-Díaz et al.^23^

**Table 1:** Specifications of the 3 (hypothetical) target trials and the 3 emulated target trials.

**Target trials**	**Emulated target trials**
*Eligibility criteria:* Pregnant individuals entering prenatal care between 2002 and 2008, from all health facilities in Norway, with an anxiety or depressive disorder diagnosed prior to pregnancy. The pregnant individuals are eligible for the early-pregnancy trial until gestational week 16, mid-pregnancy trial between gestational week 17 and gestational week 28, and the late-pregnancy trial from gestational week 29 until the end of pregnancy, given they had no previous treatment with benzodiazepines or z-hypnotics during pregnancy.	*Eligibility criteria:* MoBa participants between 2002 and 2008 who had a singleton pregnancy recorded in the MBRN and a history of anxiety or depression measured by: (a) self-reported lifetime history of major depression, and/or (b) self-reported anxiety or depression prior to pregnancy, and/or (c) self-reported use of antidepressants during the 6 months prior to pregnancy. Early-pregnancy trial: completed MoBa questionnaire 1. Eligible until gestational week 16. Mid-pregnancy trial: completed MoBa questionnaires 1 and 3, no previous treatment with benzodiazepines or z-hypnotics during pregnancy. Eligible from gestational week 17 until gestational week 28. Late-pregnancy trial: completed MoBa questionnaires 1, 3, and 4; no previous treatment with benzodiazepines or z-hypnotics during pregnancy. Eligible from gestational week 29 until the end of pregnancy.
*Treatment strategies:* 1) Benzodiazepines or z-hypnotics at enrollment, or 2) No treatment at enrollment.	*Treatment strategies:* Same as in the target trials.
*Treatment assignment:* Random assignment to the treatment strategies.	*Treatment assignment:* Defined as initiators of the benzodiazepines or z-hypnotics if the self-reported data on medication use were consistent with use in the specific trials. Assume that the groups are comparable conditional on covariates available at time 0 in each trial.
*Start and end of follow-up:* Follow-up from assignment until the children’s age of the national fifth-grade tests, death, or loss to follow-up, whichever occurs first.	*Start and end of follow-up:* Start of follow-up at gestational weeks 0, 17, and 29 in the early-, mid-, and late-pregnancy trials, respectively. End of follow-up at the children’s age of the national fifth-grade tests, death, or loss to follow-up, whichever occurs first.
*Outcomes:* The outcomes of interest are the child’s national fifth-grade test scores in numeracy and literacy.	*Outcomes:* Same as in the target trials for the early-, mid-, and late-pregnancy trials.
*Causal contrasts:* Intention-to-treat effect.	*Causal contrasts:* Observational analog of the intention-to-treat effect.
*Data analysis plan:* Intention-to-treat analysis comparing the test scores for those assigned to initiate treatment in the different trials vs those not assigned to start treatment.	*Data analysis plan:* Same as in the target trials with additional adjustments for baseline covariates by inverse probability of treatment weights using propensity scores to mimic randomization. In addition, estimate missing covariates and outcome weights and censoring weights.

Abbreviations: MBRN, Medical Birth Registry of Norway; MoBa, Norwegian Mother, Father and Child Cohort Study.

#### Eligibility criteria

Pregnant individuals entering prenatal care, from all health facilities in Norway, with an anxiety or depressive disorder diagnosed prior to pregnancy are eligible for the trials. The pregnant individuals are eligible for the early-pregnancy trial until gestational week 16, the mid-pregnancy trial between gestational week 17 and gestational week 28, and the late-pregnancy trial from gestational week 29 until the end of pregnancy, given they had no previous treatment with benzodiazepines or z-hypnotics during pregnancy.

#### Treatment strategies

Individuals are treated with benzodiazepines or z-hypnotics at enrollment or have no treatment. The treatment is used as needed and can be modified based on the symptoms of anxiety and/or depression. Both groups receive regular assessments throughout the trials.

#### Treatment assignment

The individuals are randomly assigned to treatment with benzodiazepines or z-hypnotics or no treatment at enrollment.

#### Start and end of follow-up

The individuals are followed from treatment assignment until the child completes fifth grade (generally ages 10-11).

#### Outcomes

The outcomes of interest are the child’s scholastic skills in numeracy and literacy, assessed by the national fifth-grade test scores. The national tests were introduced in 2007 in Norway as part of the national quality assessment system. All Norwegian schools are obliged to hold national tests in literacy, numeracy, and English in grades 5, 8, and 9, and the tests are mandatory. Only children with special educational or special language training needs are exempted from a test.^27^ The test scores are standardized as *z* scores over the total population of children taking the tests, separately for literacy and numeracy, for each test year.

#### Causal contrasts

Intention-to-treat effect^20^ (ie, the effect of being assigned to initiate benzodiazepine and/or z-hypnotic treatment vs no treatment) is of interest rather than the per-protocol effect because benzodiazepines and z-hypnotics are used episodically, which makes it challenging to define a treatment strategy that should be followed by individuals throughout pregnancy.

#### Data analysis plan

Intention-to-treat analysis compares the test scores for those assigned to initiate treatment with those assigned to not initiate treatment in each trial.

### Emulated target trials

To emulate the target trials, we used data from the Norwegian Mother, Father and Child Cohort Study (MoBa) linked to the Medical Birth Registry of Norway (MBRN) and Statistics Norway (SSB) via the personal identification numbers of participants. The establishment of MoBa and initial data collection were previously based on a license from the Norwegian Data Protection Authority Agency and received approval from the Regional Committees for Medical and Health Research Ethics. The MoBa cohort is regulated by the Norwegian Health Registry Act. Written informed consent was obtained from all individuals before participation in the MoBa. The current study was approved by the Regional Committees for Medical and Health Research Ethics (reference number 2017/2205).

MoBa is a population-based pregnancy cohort study conducted by the Norwegian Institute of Public Health.^28^ Participants were recruited throughout Norway from 1999 to 2008 through a postal invitation in connection with the routine ultrasound examination offered around pregnancy week 17. Forty-one percent of the invited pregnant individuals consented to participate in the MoBa. The cohort includes approximately 114 500 children, 95 200 mothers, and 75 200 fathers. Follow-up is conducted by questionnaires at regular intervals and is ongoing. This current study used data from MoBa questionnaire 1 (sent out in gestational weeks 15-17), questionnaire 3 (sent out in gestational week 30), and questionnaire 4 (sent out 6 months postpartum) and was based on version 12 of the quality-assured files released for research in 2019. The MBRN is a nationwide registry that has prospectively collected information on all births in Norway since 1967.^29^ The registry includes data on all live births, stillbirths, and abortions after gestational week 12. SSB contains information from public registries.^30^ For the present study, we acquired data on maternal educational level, family income, and children’s school test results.

We emulated the 3 target trials: the early-pregnancy trial from gestational weeks 0 to 16, the mid-pregnancy trial from gestational weeks 17 to 28, and the late-pregnancy trial from gestational week 29 to end of pregnancy. The emulated trials are identical except for the timing of enrollment and the number of eligible individuals. The reason for choosing exactly these time points for enrollment in the trials is the structure of the MoBa study (ie, completion of questionnaires 1 and 3 around gestational weeks 17-18 and 30-31, respectively) and the measurements of maternal symptoms of depression and anxiety upon completion of these questionnaires. The emulation of each component of the target trials is described below.

#### Eligibility criteria

The eligible individuals were MoBa participants between 2002 and 2008 who completed MoBa questionnaire 1, had a singleton pregnancy recorded in the MBRN, and had a history of anxiety and/or depression before pregnancy measured by (a) self-reported lifetime history of major depression (LTH of MD), (b) self-reported anxiety or depression prior to pregnancy, and/or (c) self-reported use of antidepressants during the 6 months prior to pregnancy. All participants were eligible for the early-pregnancy trial until gestational week 16. Additional eligibility criteria for the mid-pregnancy trial included the following: completed MoBa questionnaire 3 and had no previous treatment with benzodiazepines or z-hypnotics during pregnancy. Additional eligibility criteria for the late-pregnancy trial included the following: completed MoBa questionnaires 3 and 4 and had no previous treatment with benzodiazepines or z-hypnotics during pregnancy. Participants were eligible for the mid-pregnancy trial from gestational week 17 until gestational week 28 and for the late-pregnancy trial from gestational week 29.

**Figure 1:**
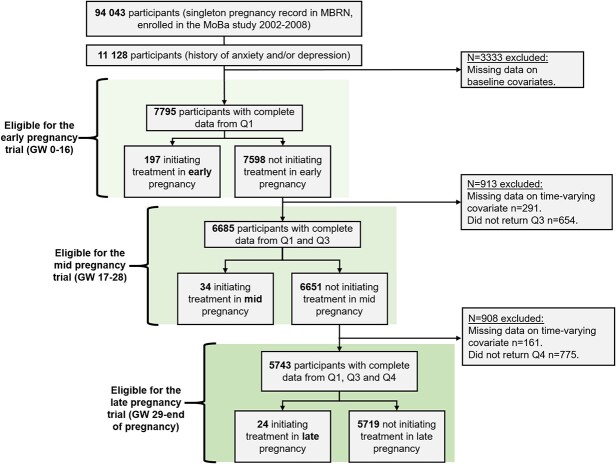
Flowchart showing the inclusion and exclusion criteria for the emulated early-, mid-, and late-pregnancy trials. MoBa children born between 1999 and 2001 were not included in this study because of lack of consent (they became 18 years before the follow-up was completed). Conditions of exclusion can overlap. Abbreviations: GW, gestational week; MBRN, medical birth registry of Norway; MoBa, Norwegian Mother, Father and Child Cohort Study; Q1, MoBa questionnaire 1; Q3, MoBa questionnaire 3; Q4, MoBa questionnaire 4.

The inclusion and exclusion criteria are presented in Figure 1.

#### Treatment strategies

The treatment strategies were treatment with benzodiazepines or z-hypnotics at enrollment or no treatment. Benzodiazepine and z-hypnotic use was self-reported by the participants in the MoBa questionnaires, according to 4-week intervals (gestational weeks 0-4, 5-8, and so on). Medications were coded according to the Anatomical Therapeutic Chemical (ATC) groups.^31^ Benzodiazepines were classified as ATC groups N05BA (benzodiazepine-anxiolytics), N05CD (benzodiazepine-hypnotics), and N03AE01 (benzodiazepine-antiepileptics). Z-hypnotics were classified as ATC group N05CF. Use of benzodiazepines and z-hypnotics in pregnancy was categorized as follows: early (gestational weeks 0-16), mid (gestational weeks 17-28), and late (gestational week 29 to end of pregnancy).

#### Treatment assignment

**Figure 2:**
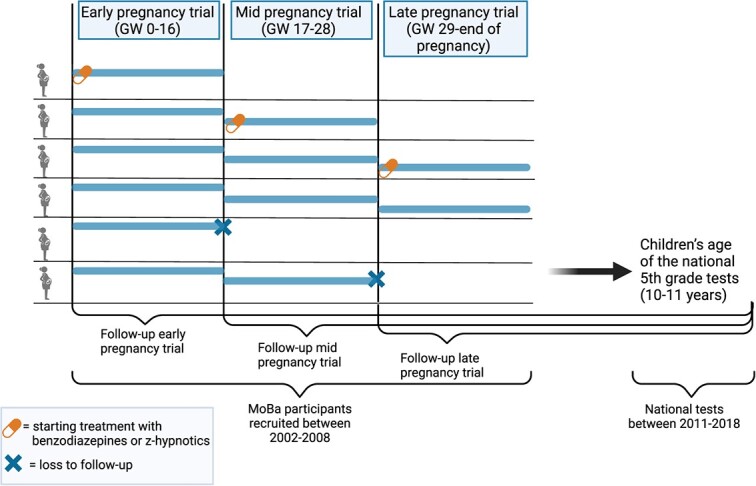
Enrollment in the early-, mid-, and late-pregnancy trial and the follow-up period. Illustrating that 1 individual can be a control in the early pregnancy trial, even if the individual initiated treatment in the mid-pregnancy trial. Created with BioRender.com.

Within each trial, we defined the eligible individuals as initiators of benzodiazepine or z-hypnotic treatment if they had self-reported data on benzodiazepine or z-hypnotic use consistent with use in that specific trial (early-pregnancy trial: gestational weeks 0-16; mid-pregnancy trial: gestational weeks 17-28; late-pregnancy trial: gestational week 29 to end of pregnancy). Eligible individuals with self-reported data on benzodiazepine or z-hypnotic use consistent with no initiation were defined as not initiating treatment within that trial (ie, individuals who had data compatible with treatment initiation in late pregnancy were defined as not initiating treatment in both the early and mid-pregnancy trials) (Figure 2).

In each trial, the eligible individuals were not randomly assigned to the treatment strategies as in the target trial, and thus we assumed that the groups were comparable conditional on the baseline covariates for each trial.^20^ Potential confounders were identified through the literature and directed acyclic graphs (Figure S1).^32^^,^^33^ The baseline covariates were the following, characterized as described in Table 2: maternal age, parity, smoking, prepregnancy body mass index, alcohol use, marital status, education, family income-to-needs ratio (1 year before the child was born), sleeping problems, chronic diseases, self-reported anxiety, depression and sleeping problems, and lifetime history of major depression. In addition, some covariates were measured at different time points during pregnancy, and thus the baseline covariates adjusted for in the different emulated target trials varied. Maternal symptoms of depression and anxiety, assessed with a validated short version of the Hopkins Symptom Checklist,^34^ were measured at gestational weeks 17 and 30. In addition, comedication use (nonsteroidal anti-inflammatory drugs, opioids, paracetamol, antidepressants, antipsychotics, and antiepileptics) was measured during the 6 months prior to pregnancy, early pregnancy, and mid-pregnancy. Information on covariates that were available at time 0 for each trial was included as baseline covariates (Figures S2-S4). Appendix S1 provides further information.

**Table 2:** Characteristics of individuals with complete data according to treatment initiation with benzodiazepines or z-hypnotics in each trial.

**Characteristic**	**Individuals eligible for the early-pregnancy trial, *n* = 7795, No. (%)**		**Individuals eligible for the mid-pregnancy trial, *n* = 6685, No. (%)**		**Individuals eligible for the late-pregnancy trial, *n* = 5743, No. (%)**	
	**Treatment initiation in early pregnancy**	**No treatment initiation in early pregnancy**	**Treatment initiation in mid-pregnancy**	**No treatment initiation in mid-pregnancy**	**Treatment initiation in late pregnancy**	**No treatment initiation in late pregnancy**
No. of participants	197 (2.5)	7598 (97.5)	34 (0.5)	6651 (99.5)	24 (0.4)	5719 (99.6)
Age						
<25	27 (13.7)	1109 (14.6)	4 (11.8)	940 (14.1)	3 (12.5)	755 (13.2)
25-29	48 (24.4)	2363 (31.1)	5 (14.7)	2090 (31.4)	6 (25.0)	1833 (32.0)
30-34	69 (35.0)	2710 (35.7)	19 (55.9)	2381 (35.8)	9 (37.5)	2057 (36.0)
≥35	53 (26.9)	1416 (18.6)	6 (17.6)	1240 (18.6)	6 (25.0)	1074 (18.8)
Parity						
Primiparous	89 (45.2)	3619 (47.6)	10 (29.4)	3217 (48.4)	12 (50.0)	2808 (49.1)
Multiparous	108 (54.8)	3979 (52.4)	24 (70.6)	3434 (51.6)	12 (50.0)	2911 (50.9)
Marital status						
Married/cohabitant	169 (85.8)	7107 (93.5)	33 (97.1)	6257 (94.1)	22 (91.7)	5389 (94.2)
Other	28 (14.2)	491 (6.5)	1 (2.9)	394 (5.9)	2 (8.3)	330 (5.8)
Prepregnancy BMI						
<18.5	13 (6.6)	294 (3.9)	4 (11.8)	247 (3.7)	2 (8.3)	200 (3.5)
18.5-24.9	116 (58.9)	4709 (62.0)	17 (50.0)	4119 (61.9)	13 (54.2)	3565 (62.3)
25.0-29.9	46 (23.4)	1674 (22.0)	9 (26.4)	1486 (22.3)	5 (20.8)	1285 (22.5)
≥30	22 (11.1)	921 (12.1)	4 (11.8)	799 (12.1)	4 (16.7)	669 (11.7)
Alcohol^a^						
No	185 (93.9)	7320 (96.3)	31 (91.2)	6420 (96.5)	23 (95.8)	5520 (96.5)
Yes	12 (6.1)	278 (3.7)	3 (8.8)	231 (3.5)	1 (4.2)	199 (3.5)
Smoking^b^						
No	132 (67.0)	6341 (83.5)	29 (85.3)	5602 (84.2)	22 (91.7)	4854 (84.9)
Yes	65 (33.0)	1257 (16.5)	5 (14.7)	1049 (15.8)	2 (8.3)	865 (15.1)
Educational level^c^						
10-year primary school or less	45 (22.8)	1048 (13.8)	6 (17.6)	847 (12.7)	—	647 (11.3)
Secondary/vocational school	64 (32.5)	2330 (30.7)	9 (26.5)	2047 (30.8)	9 (37.5)	1726 (30.2)
College or advanced degree	88 (44.7)	4220 (55.5)	19 (55.9)	3757 (56.5)	15 (62.5)	3346 (58.5)
Family income, ITNR^d^						
<2	102 (51.8)	3367 (44.3)	13 (38.2)	2895 (43.5)	6 (25.0)	2435 (42.6)
≥2	95 (48.2)	4231 (55.7)	21 (61.8)	3756 (56.5)	18 (75.0)	3284 (57.4)
Anxiety prepregnancy	122 (61.9)	2203 (29.0)	21 (61.8)	1906 (28.7)	14 (58.3)	1611 (28.2)
Depression prepregnancy	141 (71.6)	4196 (55.2)	24 (70.6)	3662 (55.1)	17 (70.8)	3129 (54.7)
Sleeping problems in early pregnancy	110 (55.8)	2115 (27.8)	10 (29.4)	1809 (27.2)	9 (37.5)	1529 (26.7)
LTH of MD	84 (42.6)	4075 (53.6)	16 (47.1)	3549 (53.4)	14 (58.3)	3068 (53.6)
Chronic disease prepregnancy^e^	61 (31.0)	1382 (18.2)	6 (17.6)	1195 (18.0)	6 (25.0)	1002 (17.5)
Benzodiazepine and/or z-hypnotic use during the 6 months prior to pregnancy	102 (51.8)	214 (2.8)	7 (20.6)	180 (2.7)	4 (16.7)	149 (2.6)
Symptoms of depression/anxiety at GW 17,^f^ mean (SD)			0.9 (1.2)	0.8 (1.5)	1.4 (1.9)	0.8 (1.4)
Symptoms of depression/anxiety at gestational week 30,^f^ mean (SD)			—	—	2.3 (2.2)	0.7 (1.4)

**Table 2:** Continued

**Characteristic**	**Individuals eligible for the early-pregnancy trial, *n* = 7795, No. (%)**		**Individuals eligible for the mid-pregnancy trial, *n* = 6685, No. (%)**		**Individuals eligible for the late-pregnancy trial, *n* = 5743, No. (%)**	
	**Treatment initiation in early pregnancy**	**No treatment initiation in early pregnancy**	**Treatment initiation in mid-pregnancy**	**No treatment initiation in mid-pregnancy**	**Treatment initiation in late pregnancy**	**No treatment initiation in late pregnancy**
Comedication use during the 6 months prior to pregnancy						
NSAIDs	32 (16.2)	965 (12.7)	3 (8.8)	874 (13.1)	4 (16.7)	762 (13.3)
Opioids	13 (6.6)	184 (2.4)	2 (5.9)	160 (2.4)	—	138 (2.4)
Paracetamol	62 (31.5)	2279 (30.0)	10 (29.4)	2023 (30.4)	9 (37.5)	1750 (30.6)
Antidepressants	69 (35.0)	909 (12.0)	10 (29.4)	793 (11.9)	6 (25.0)	678 (11.9)
Antipsychotics	13 (6.6)	61 (0.8)	—	53 (0.8)	—	45 (0.8)
Antiepileptics^g^	5 (2.5)	59 (0.8)	—	48 (0.7)	—	37 (0.6)
Comedication use in early pregnancy						
NSAIDs			3 (8.8)	460 (6.9)	—	397 (6.9)
Opioids			5 (14.7)	117 (1.8)	—	99 (1.7)
Paracetamol			23 (67.6)	2831 (42.6)	13 (54.2)	2426 (42.4)
Antidepressants			12 (35.3)	530 (8.0)	2 (8.3)	450 (7.9)
Antipsychotics			3 (8.8)	86 (1.3)	—	72 (1.3)
Antiepileptics^g^			—	45 (0.7)	—	36 (0.6)
Comedication use in mid-pregnancy						
NSAIDs					—	89 (1.6)
Opioids					2 (8.3)	69 (1.2)
Paracetamol					8 (33.3)	1580 (27.6)
Antidepressants					3 (12.5)	165 (2.9)
Antipsychotics					—	23 (0.4)
Antiepileptics^g^					—	6 (0.1)

Abbreviations: BMI, body mass index; GW, gestational week; ITNR, income-to-needs ratio; LTH of MD, lifetime history of major depression; NSAID, nonsteroidal anti-inflammatory drug.

^a^Alcohol use was measured in MoBa questionnaire 1.

^b^Smoking was measured in early pregnancy.

^c^Educational level assessed in the child’s birth year.

^d^Family income was assessed by ITNR.

^e^Chronic disease included asthma, diabetes, hypertension, epilepsy, arthritis, other heart disease, thyroid disorder, lupus, or Crohn disease, reported before pregnancy.

^f^Presence of symptoms of depression or anxiety indicated on the 5-item short version of the Hopkins Symptoms Checklist (SCL-5).

^g^Antiepileptic use does not include clonazepam (N03AE01).

#### Start and end of follow-up

The follow-up is the same as in the target trials.

#### Outcomes

The outcomes are the same as in the target trials. The raw tests scores were compared between the MoBa population and the total population of children taking the tests (Table S1).

#### Causal contrast of interest

The causal contrast of interest is the observational analog of the intention-to-treat effects.^20^

#### Statistical analysis

The statistical analysis is the same as in the target trials with additional adjustments for baseline covariates by stabilized inverse probability of treatment weights (IPTWs) using the propensity score.^35^^‑^^37^ For each trial, the propensity scores were estimated with a logistic regression model estimating the probability of benzodiazepine or z-hypnotic use in that trial for each individual, conditional on baseline covariates.^37^ To assess the balance of baseline covariates between exposed and unexposed groups, we calculated standardized mean and proportion differences.^38^ A difference less than 0.1 was considered negligible.^37^ Covariates that remained imbalanced after weighting were included in the outcome model.

In addition, we derived weights to address missingness in covariates and outcomes, as well as censoring weights to address loss to follow-up between completing the MoBa questionnaires. Missing covariate weights were estimated to account for missing data on baseline covariates in each trial. The probability of having complete data on the covariates in each trial was estimated using logistic regression models. Only covariates with complete information were included in the model. We estimated stabilized inverse probability of missing covariate weights, such that individuals who had complete data were up-weighted to represent similar individuals who were excluded because of missing covariate data.^39^^,^^40^ The missing covariate weights were included in the propensity score models.

We estimated missing outcome weights to adjust for some individuals’ missing information. The probability of having outcome data (separately for literacy and numeracy) was estimated using logistic regression models, weighted by the missing covariates weights. We estimated stabilized inverse probability of missing outcome weights, such that individuals with outcome data were up-weighted to represent similar individuals with missing outcome data.^39^^‑^^41^

Lastly, we estimated censoring weights to account for MoBa questionnaires not returned. The probability of returning the questionnaires in the mid- and late-pregnancy trials was estimated using logistic regression, weighted by the missing covariates weights. Stabilized inverse probability of censoring weights (IPCWs) were estimated to up-weight individuals who completed MoBa questionnaires 3 and 4 to represent similar individuals who dropped out.^42^^‑^^44^

Table S2 provides detailed information on the specification of the different weights.

The stabilized IPTWs, stabilized inverse probability of missing data weights (for covariates and outcome), and stabilized IPCWs were combined to a final stabilized weight for each individual in each trial.^41^^,^^45^  Table S3 describes how the weights were combined. Linear outcome models were fitted to estimate crude mean differences (Δ_c_) and weighted mean differences (Δ_w_) in standardized test scores separately for numeracy and literacy, with 95% confidence intervals obtained by bootstrapping (15 000 replications) using the bias-corrected and accelerated (BCa) bootstrap method.^46^ The weights were reestimated in each bootstrap replicate.

#### Sensitivity analyses

We performed several sensitivity analyses to assess the robustness of our findings. First, the eligibility criteria in each trial were changed to include only pregnant individuals who did not use benzodiazepines or z-hypnotics during the 6-month period prior to pregnancy. The rationale for changing the eligibility criteria is that the pregnant individuals assigned to initiate benzodiazepine and z-hypnotics in these trials represent “new users.”^20^^,^^47^ Second, we specified 3 target trials capturing all pregnant individuals regardless of a history of anxiety or depression prior to pregnancy to allow anyone potentially at risk of experiencing new-onset anxiety or depression during pregnancy. The specifications of these trials are identical to the trials already described, except for the eligibility criteria, which now include all pregnant individuals, without restricting to those with a history of anxiety and/or depression prior to pregnancy (Figure S5). Third, we specified target trials allowing new cases of anxiety and/or depression in early and mid-pregnancy to be eligible in the subsequent trials (Appendix S1). Fourth, we specified target trials of discontinuation in early pregnancy with and without the eligibility criteria requiring a history of anxiety and/or depression prior to pregnancy (Appendix S1). Lastly, we pooled the trials in the main analysis.

When interpreting the results, we chose to focus on the magnitude of the estimates and the width of the 95% confidence intervals rather than dichotomizing the results as statistically significant or not.^48^

All statistical analyses were performed with R (version 4.0.3; R Foundation for Statistical Computing). Data were analyzed from September 2022 to November 2023.

## Results

A total of 7795 individuals were eligible for the early-pregnancy trial, of whom 197 (2.5%) initiated treatment with benzodiazepines or z-hypnotics. In total, 6685 were eligible for the mid-pregnancy trial (34 [0.5%] initiated treatment), and 5743 were eligible for the late-pregnancy trial (24 [0.4%] initiated treatment) (Figure 1; Table S4). Benzodiazepine-anxiolytics and z-hypnotics were the most commonly used groups (Table S4). Characteristics of the pregnant individuals eligible for each trial are presented in Table 2. Individuals who initiated benzodiazepine or z-hypnotic treatment in any of the trials were more likely to be older, smoke and drink alcohol, and use more concomitant medications. The complete and the incomplete samples did not differ substantially (Table S5). Between 0.1% and 0.3% of the individuals eligible for the trials gave birth before gestational week 29 (Table S6).

The test results presented as *z* scores were compared between the total MoBa population and the eligible individuals in each trial (Table S7). Approximately 12% to 13% of the children in the trials had numeracy or literacy test scores 1 SD below the population mean. On all tests, between 8% and 10% of children born to eligible individuals were missing information on at least 1 test score (Table S8). A total of 25 (0.3%) children in the early-pregnancy, 18 (0.3%) in the mid-pregnancy, and 6 (0.1%) in the late-pregnancy trials were excluded because they died during follow-up. Exemptions from the tests were given for children with special educational or special language training needs: between 0.3% and 0.4% of the children were exempted from the numeracy tests, and 0.7% to 0.8% were exempted from the literacy tests (Figure S6). Only 2 of the children exempted from the literacy tests were born to mothers initiating treatment with benzodiazepines or z-hypnotics in the early- and late-pregnancy trials, respectively, and 1 child born to an initiator in the late-pregnancy trial was exempted from the numeracy test (Figure S6). Mothers of children without outcome information have slightly lower educational level and family income and higher prevalence of chronic disease and LTH of MD than the mothers of children with outcome information (Tables S9-S10). Characteristics of the final weights are shown in Table S11 (separately for the IPTWs in Table S12). Balance of baseline covariates between the individuals initiating treatment in pregnancy vs not initiating treatment is presented in Figures S7 to S9.

In both the crude and weighted analyses, children of mothers initiating treatment with benzodiazepines or z-hypnotics in the mid- and late-pregnancy trials scored lower on tests in literacy compared to children of mothers not initiating treatment in the corresponding trials (mid: Δ_w_, –0.46 [95% CI, –1.18 to 0.12]; late: Δ_w_, –0.36 [95% CI, –1.71 to 0.32]). We observed no difference in literacy test scores for children of mothers initiating treatment in the early-pregnancy trial compared to children of mothers not initiating treatment. For the test in numeracy, we did not observe any differences in test scores for children of mothers initiating treatment in the early- or late-pregnancy trials compared to children of mothers not initiating treatment in the corresponding trials (Table 3). Children of mothers initiating treatment with benzodiazepines or z-hypnotics in the mid-pregnancy trial scored lower on tests in numeracy compared to children of mothers not initiating treatment in the mid-pregnancy trial (Δ_w_, –0.26 [95% CI, –0.70 to 0.99]). The 95% CIs included the null for all analyses.

**Table 3:** Crude and weighted mean differences in the children’s test scores in literacy and numeracy according to the mothers’ initiation of benzodiazepines or z-hypnotics in each trial (early-pregnancy trial, *N* = 7795; mid-pregnancy trial, *N* = 6685; late-pregnancy trial, *N* = 5743).

	***N***	***z* score, mean (SD)**	**Crude mean difference (95% CI)**	**Weighted mean difference (95% CI)**
**Literacy**				
Treatment initiation in the **early-**pregnancy trial	197	0.17 (0.9)	–0.04 (–0.18 to 0.10)	–0.03 (–0.26 to 0.19)
No treatment initiation in the **early-**pregnancy trial	7598	0.21 (1.0)		
Treatment initiation in the **mid-**pregnancy trial	34	–0.07 (1.0)	–0.30 (–0.66 to 0.07)	–0.46 (–1.18 to 0.12)
No treatment initiation in the **mid-**pregnancy trial	6651	0.23 (1.0)		
Treatment initiation in the **late-**pregnancy trial	24	0.10 (1.0)	–0.15 (–0.64 to 0.21)	–0.36 (–1.71 to 0.32)
No treatment initiation in the **late-**pregnancy trial	5719	0.25 (1.0)		
**Numeracy**				
Treatment initiation in the **early-**pregnancy trial	197	0.04 (1.0)	–0.11 (–0.26 to 0.04)	–0.05 (–0.36 to 0.26)
No treatment initiation in the **early-**pregnancy trial	7598	0.15 (1.0)		
Treatment initiation in the **mid-**pregnancy trial	34	–0.13 (0.9)	–0.30 (–0.60 to 0.05)	–0.26 (–0.70 to 0.99)
No treatment initiation in the **mid-**pregnancy trial	6651	0.17 (1.0)		
Treatment initiation in the **late-**pregnancy trial	24	0.18 (1.1)	–0.01 (–0.48 to 0.45)	0.11 (–0.50 to 0.93)
No treatment initiation in the **late-**pregnancy trial	5719	0.19 (1.0)		

### Sensitivity analysis

**Figure 3:**
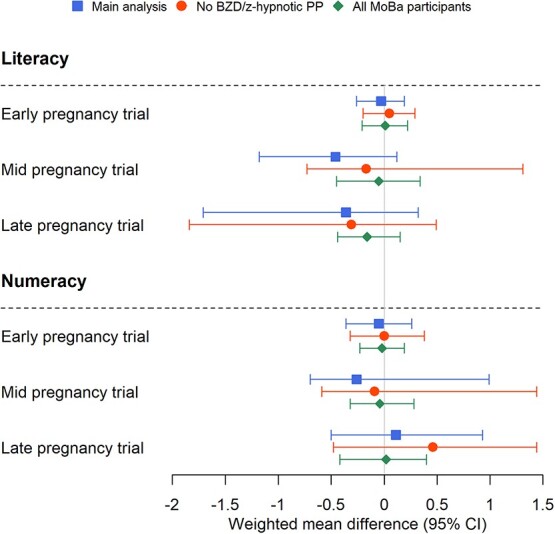
Weighted mean differences in test scores in literacy and numeracy for children of mothers initiating treatment with benzodiazepines or z-hypnotics compared to children of noninitiators, in each trial, for the main analysis (blue square) and the sensitivity analyses (restricting to individuals with no treatment during the 6 months prepregnancy [red circle] and all MoBa participants without requiring a history of anxiety and/or depression [green diamond]). Abbreviations: BZD, benzodiazepine; MoBa, Norwegian Mother, Father and Child Cohort Study; pp, prepregnancy.

Changing eligibility criteria to exclude individuals with prepregnancy benzodiazepine or z-hypnotic use produced results that were comparable to the main analysis with some attenuation of estimates for the mid-pregnancy trial (literacy: sensitivity analysis Δ_w_, –0.17 [95% CI, –0.73 to 1.31]; main analysis Δ_w_, –0.46 [95% CI, –1.18 to 0.12]; numeracy: sensitivity analysis Δ_w_, –0.09 [95% CI, –0.59 to 1.44]; main analysis Δ_w_, –0.26 [95% CI, –0.70 to 0.99]) (Figure 3; Table S13).

For the trials including all pregnant individuals in the MoBa, without requiring a history of anxiety or depression prior to pregnancy, a total of 67 280 individuals were eligible for the early-pregnancy trial, in which 370 (0.6%) individuals initiated treatment, 60 820 were eligible for the mid-pregnancy trial (94 [0.2%] initiated treatment), and 54 400 were eligible for the late-pregnancy trial (74 [0.1%] initiated treatment) (Figure S5). Characteristics of the pregnant individuals eligible for each trial are presented in Table S14. The results from these trials were similar to the results from the main trials. We did not observe any differences in numeracy test scores for children of mothers initiating treatment with benzodiazepines or z-hypnotics in any of the trials compared to children of mothers not initiating treatment. For literacy test scores, we did not observe any differences between children of mothers initiating treatment compared to children of mothers not initiating treatment in the early- and mid-pregnancy trials. The results showed attenuated differences between children of mothers initiating treatment in the late-pregnancy trial compared to the main analysis (sensitivity analysis: Δ_w_, –0.16 [95% CI, –0.44 to 0.15]; main analysis: Δ_w_, –0.36 [95% CI, –1.71 to 0.32]; Figure 3; Table S15).

The analysis allowing new cases of anxiety and/or depression in early and mid-pregnancy to be eligible in the subsequent trials showed attenuated differences in children’s literacy test scores compared to the main analysis, while for numeracy test scores, the results were similar to the main analysis (Table S16). For the discontinuation analysis, results indicate no differences in literacy test scores for children of mothers continuing treatment with benzodiazepines and/or z-hypnotics in early pregnancy compared to children of mothers discontinuing treatment. For the test in numeracy, children of mothers continuing treatment in early pregnancy scored lower compared to children of mothers discontinuing treatment (Tables S17 and S18). Pooled results indicate no differences in numeracy or literacy test scores between children of mothers initiating treatment in pregnancy and children of mothers not initiating treatment in pregnancy (Table S19).

## Discussion

In this study, we emulated 3 trials to evaluate the effect of timing of treatment initiation with benzodiazepines or z-hypnotics during pregnancy on children’s scholastic skills. We found that children of mothers who initiated treatment in mid-pregnancy showed lower scholastic skills in literacy and numeracy compared to children of mothers not initiating treatment in mid-pregnancy, in addition to lower scholastic skills in literacy for children of mothers initiating treatment in late pregnancy compared to children of noninitiators. However, we found no difference in test scores in literacy for treatment initiation vs no treatment initiation in the early-pregnancy trial, or for initiation in any early or late pregnancy, and test scores in numeracy. For all analyses, the differences in mean standardized test scores were small; the 95% confidence intervals were wide and included the null and should thus be interpreted with caution.

To our knowledge, this is one of the first studies to examine scholastic skills in children exposed to benzodiazepines or z-hypnotics during fetal life. A Norwegian study using MoBa data found no increased risk of lower language competence at 3 years of age associated with prenatal exposure to benzodiazepines and z-hypnotics.^49^ A Danish registry study investigated the association between prenatal exposure to clonazepam, based on maternal prescription fills, and school performance in Danish in grades 2, 4, 6, and 8 and mathematics in grades 3 and 6.^15^ The results showed signs of impaired school performance associated with prenatal clonazepam exposure (Danish in grade 6, mean difference: –0.07, 95% CI, –0.13 to –0.02; mathematics in grade 3, mean difference: –0.06, 95% CI, –0.11 to –0.01); however, the findings were not consistent, and the absolute differences were small.^15^ The results from the current study are in line with these prior studies.

### Limitations

This study had some limitations. First, the medication use and symptoms of depression and anxiety were self-reported by the MoBa study participants, but this may still be an improvement over registry-based studies, which have no information on symptom severity. In addition, symptoms of depression and anxiety were not measured around conception and therefore not included as a baseline covariate for the analysis of the early-pregnancy trial. Thus, we cannot rule out residual confounding by severity of depression and anxiety. The eligibility criteria did not fully capture all indications for use of benzodiazepines and z-hypnotics, potentially affecting the generalizability of the findings beyond the target population. Second, MoBa has a low response rate (41%) and might not represent the general population.^50^ In general, MoBa participants are wealthier and have higher educational attainment than the general population.^50^ This study uses population-based testing for the outcome rather than parent-reported questionnaires, which are subject to significantly greater loss to follow-up.^51^ Third, for several analyses, we had low study power, and the numbers of initiators of treatment with benzodiazepines or z-hypnotics in the mid- and late-pregnancy trials were low. We did not have enough power to study separate benzodiazepine groups or z-hypnotics alone. Fourth, to set the eligibility criteria for each trial, we had to condition on the participants’ completion of the MoBa questionnaires in the future, in addition to having a pregnancy recorded in the MBRN, which could have introduced bias by selection of pregnancies that lasted until the criteria for eligibility were measured. However, this may be an improvement over prior studies that required live birth or completion of all questionnaires. For the early-pregnancy trial, we could have competing risks with pregnancy losses.^23^ Some studies have reported associations between benzodiazepine and z-hypnotic use during pregnancy and spontaneous abortions,^52^ but it remains unclear whether these associations are causal. Lastly, we did not take into account that some individuals gave birth before they could potentially be eligible for the late-pregnancy trial, although this applies to only a few participants.

## Conclusion

Using an emulated trial approach and population-based scholastic tests, the results of this study did not show any effects of mothers initiating treatment with benzodiazepines or z-hypnotics at different time points during pregnancy on the children’s fifth-grade standardized test scores in numeracy and literacy. The results provide reassurance for patients in need of benzodiazepines and z-hypnotics during pregnancy; however, the findings must be interpreted with caution due to low study power in some of the analyses.

## Supplementary Material

Web_Material_kwae159

## Data Availability

Data from the Norwegian Mother, Father and Child Cohort Study and the Medical Birth Registry of Norway used in this study are managed by the national health register holders in Norway (Norwegian Institute of Public Health) and can be made available to researchers, provided approval from the Regional Committees for Medical and Health Research Ethics (REC), compliance with the EU General Data Protection Regulation (GDPR), and approval from the data owners. The consent given by the participants does not allow the storage of data on an individual level in repositories or journals. Researchers who want access to data sets for replication should apply through helsedata.no. Access to data sets requires approval from the Regional Committee for Medical and Health Research Ethics in Norway and an agreement with MoBa.
