# Supplementary material for: Prenatal exposure to benzodiazepine and z-hypnotics and fifth-grade scholastic skills—emulating target trials using data from the Norwegian Mother, Father and Child Cohort Study
Source: Am J Epidemiol. 2024 Jun 29;194(1):73–84. doi: 10.1093/aje/kwae159 (PMC11735967; doi:10.1093/aje/kwae159)
Supplement: Web_Material_kwae159 [file web_material_kwae159.docx]

## **Supplementary Material**

Prenatal exposure to benzodiazepines and z-hypnotics and fifth-grade scholastic skills – emulating target trials using data from the Norwegian Mother, Father and Child Cohort Study.

Authors: Lene Maria Sundbakk, Mollie Wood, Jon Michael Gran, Hedvig Nordeng

**Table of contents:**

**Appendix S1**

- Study sample
- Outcomes
- Covariates
- Sensitivity analyses

**Table S1**. Test scores among children in MoBa and all children in Norway who took the test, according to subject and test year.

**Table S2.** Description of the model specifications for the different inverse probability weights (IPWs).

**Table S3.** Specification of the final combined stabilized weights used in each analysis.

**Table S4.** Use of benzodiazepines and z-hypnotics in the different time window.

**Table S5.** Characteristics of individuals with incomplete data according to treatment initiation with benzodiazepines or z-hypnotics in each trial.

**Table S6.** Overview of the gestational ages at birth for the eligible individuals in each trial.

**Table S7.** National test results of the MoBa children presented as z-scores, by the mothers’ initiation status in each trial.

**Table S8.** Overview of the number of children with missing information on the outcomes, according to the mothers’ eligibility to the trials.

**Table S9.** Characteristics of individuals in each trial according to whether outcome information was available for the numeracy test.

**Table S10**. Characteristics of individuals in each trial according to whether outcome information was available for the literacy test.

**Table S11**. Characteristics of the final combined stabilized IPWs.

**Table S12.** Characteristics of the stabilized IPTWs.

**Table S13.** Results from sensitivity analysis, changing the eligibility criteria in each trial to include only individuals who did not use benzodiazepines and/or z-hypnotics during the 6 months prior to pregnancy.

**Table S14.** Characteristics of individuals with complete data according to treatment initiation with benzodiazepines or z-hypnotics, for the trials in the sensitivity analysis not restricting to individuals with a history of anxiety and/or depression.

**Table S15.** Results from sensitivity analysis, changing the eligibility criteria to include all MoBa participants without requiring a history of anxiety and/or depression.

**Table S16.** Results from sensitivity analysis, for new cases of anxiety and/or depression in early or mid pregnancy.

**Table S17.** Results from sensitivity analysis: Continuation vs. discontinuation in early pregnancy among women who used benzodiazepines or z-hypnotics during the 6 months prior to pregnancy.

**Table S18.** Results from sensitivity analysis: Continuation vs. discontinuation in early pregnancy among women who used benzodiazepines or z-hypnotics during the 6 months prior to pregnancy and have a history of anxiety and/or depression prior to pregnancy.

**Table S19.** Results from sensitivity analyses, pooling the trials in the main analysis.

**Figure S1.** Directed acyclic graph showing assumed covariate structure, drawn as a time-fixed model for simplicity.

**Figure S2**. DAG for the early pregnancy trial.

**Figure S3**. DAG for the mid pregnancy trial.

**Figure S4**. DAG for the late pregnancy trial.

**Figure S5.** Flowchart showing the inclusion and exclusion criteria for the emulated early, mid and late pregnancy trials in the sensitivity analysis, changing the eligibility criteria to include all MoBa participants without requiring a history of anxiety or depression.

**Figure S6.** Overview of the number of exempted children in each subject, according to their mothers’ treatment initiation status in each trial.

**Figure S7.** Balance before and after IPTW of covariates between individuals who initiated treatment vs. not initiated treatment in the early pregnancy trial.

**Figure S8**. Balance before and after IPTW of covariates between individuals who initiated treatment vs. not initiated treatment in the mid pregnancy trial.

**Figure S9**. Balance before and after IPTW of covariates between individuals who initiated treatment vs. not initiated treatment in the late pregnancy trial.

**Appendix S1**

**Study sample**

The Norwegian Mother, Father and Child Cohort Study (MoBa) recruited pregnant women throughout Norway from 1999-2008. However, because the oldest children born between 1999-2001 turned 18 before the end of follow-up in 2018, they could not be included in this study due to lack of consent (N=5828).

**Outcomes**

The national tests were introduced in 2007 in Norway, as part of the national quality assessment system (1). All Norwegian schools are obliged to hold national tests in reading/literacy, numeracy and English in 5^th^, 8^th^ and 9^th^ grade. In this study, we chose to focus on reading/literacy and numeracy. The purpose of the national tests are to generate objective knowledge about the children’s scholastic skills, in order to identify children that perform below their peers, and to follow changes in results over time (2). The tests in reading and numeracy have been found to have good reliability; 0.86 and 0.91, respectively (3). The tests are mandatory, but since the tests are done once at each school, with no re-tests, there are some students that do not meet or participate in the tests. Only children with special educational or special language training needs are exempted from a test. The students perform the tests on computers and have 90 minutes to complete one test. A comparison of the scores in each domain and each test year for MoBa participants and the study samples are showed in Table S1. A description of what is being tested in each domain follows below.

***Literacy (1):*** Reading and language is arguably the most important academic skill learned in school. The reading test is done by presenting the student with a text (roughly an A4 sheet 12pt text) and then asking multiple choice questions in different complexity about the text. This measures speed of reading as well as the ability to decompose a longer segment of coherent text. The test is designed to test for the following abilities: to find information in a text, to interpret a text, and to reflect and evaluate the content of a text. While volume training and exposure are important for most scholastic skills, this is especially important for reading. Volume training by reading different materials and books of increasing complexity is the primary way of excelling in reading and reflecting over writing content. The raw test scores range between a minimum of 0 points to a maximum of 34 points.

***Numeracy (1)***: Unlike reading, high volume and exposure is not sufficient for excelling in mathematics, with large volume being potentially detrimental to performance. The test is designed to reflect multiple sub-domains in mathematics, such as numeracy, geometry, and statistics. Numeracy is the basics of number manipulation, but is limited to addition, subtraction, multiplication, and division. Geometry and measurements, are the two and three dimensional representation of numeracy, where comparing volumes and surfaces of different measurements are evaluated. Statistics is operationalized as a visualization of data, mostly into charts and diagrams, with the addition of data summary, such as mean, median, and frequency tables. The raw test scores range between a minimum of 0 points to a maximum of 45 points.

**Covariates**

The baseline covariates included both maternal sociodemographic and medical characteristics. Data on maternal age, parity, smoking status at the start of the pregnancy were retrieved from the MBRN. Data on maternal education and family income were retrieved from the SSB. Family income was assessed by income-to-needs-ratio (ITNR), calculated by dividing the family income after tax by the poverty income threshold i.e. consumption equivalents, for a household of that size in the given year, in this case the year before the child was born. The EU-60 standard was applied (4). ITNR=1 indicates that the family is living at the poverty level, higher values indicate greater income. ITNR was grouped into 2 levels (<2, ≥2).

The MoBa provided data on pre-pregnancy body mass index (BMI), alcohol use in early pregnancy, marital status, sleeping problems in early pregnancy, chronic diseases before pregnancy, self-reported anxiety or depression before pregnancy and lifetime history of major depression (LTH of MD). The mother’s LTH of MD was reported according to five key depressive symptoms, which corresponded closely to the DSM-III criteria for lifetime major depression (5). Maternal symptoms of depression and anxiety during pregnancy and maternal co-medication use were measured at different time points during pregnancy. Maternal symptoms of depression and anxiety during pregnancy were assessed with a validated short version of the Hopkins Symptom Checklist (SCL-5) (6) at gestational week 17 and 30. Mean summary scores were calculated and standardized to z-scores at each time point. The women reported a number of concomitant medications in MoBa questionnaires 1, 3 and 4: nonsteroidal anti-inflammatory drugs (NSAIDs; ATC code M01A), opioids (ATC code N02A), paracetamol (ATC code N02BE01), antidepressants (ATC code N06A), antipsychotics (ATC code N05A) and antiepileptics (ATC code N03A). Comedications were categorized as occurring in early, mid or late pregnancy, or during the 6 months prior to pregnancy.

Below follows a description of the instrument used in the analyses. An instrument documentation is available at <https://www.fhi.no/en/studies/moba/for-forskere-artikler/questionnaires-from-moba/>.

Severity of depression and anxiety symptoms: The Hopkins Symptoms Checklist-25 (SCL-25) is a psychometric scale designed to measure symptoms of anxiety and depression in population surveys (6). SCL-5 is highly correlated to the SCL-25 (7,8). The SCL-5 composes of the following items: “Nervousness or shakiness inside”, “Feeling fearful”, “Feeling hopeless about the future”, “Feeling blue” and “Worrying too much about things”. Women could indicate whether they had been bothered by any of the listed symptoms during the last two weeks in a four-category response, from "not bothered" to "very bothered", which are rated 1 to 4, respectively.

**Sensitivity analyses**

In sensitivity analyses we specified target trials allowing new cases of anxiety and/or depression in early and mid pregnancy to be eligible in the subsequent trials. The rationale behind including this analysis is that we want to define an inclusion criteria that includes history of anxiety and/or depression by time zero of each trial, which would allow new cases of anxiety or depression during pregnancy to be eligible for the subsequent trials. The only difference between these trials and the trials in the main analysis is the eligibility criteria.

The eligibility criteria for the mid pregnancy trial are the following:

- No use of benzodiazepines or z-hypnotics during early pregnancy.
- Experience depression or anxiety during early pregnancy, defined by either:
- Depression in early pregnancy
- Anxiety in early pregnancy
- Exposure to antidepressants (N06A) in early pregnancy
- SCL5_Q1 score ≥ 2

The eligibility criteria for the late pregnancy trial are the following:

- No use of benzodiazepines in early or mid pregnancy.
- Experience depression or anxiety during mid pregnancy defined by either:
  - Depression in mid pregnancy
  - Exposure to antidepressants (N06A) in mid pregnancy
  - SCL5_Q3 score ≥ 2
  - Other psychiatric problems in mid pregnancy

We also specified target trials of discontinuation in early pregnancy; one requiring a history of anxiety and/or depression and the other did not require a history of anxiety or depression. The eligibility criteria are otherwise identical to the trials in the main analysis.

**References**

1. The Norwegian Directorate for Education and Training. Kva er nasjonale prøver? Accessed January 18, 2023. <https://www.udir.no/eksamen-og-prover/prover/nasjonale-prover/om-nasjonale-prover/>

2. The Norwegian Directorate for Education and Training. Primary, Lower and Upper Secondary School. Accessed January 18, 2023. <https://www.udir.no/in-english/quality-in-ecec-schools-and-vocationa-education-and-training/kindergarden/>

3. Utdanningsdirektoratet. Nasjonale prøver 2013 - fagmiljøenes analyse av prøvene. Updated February 4, 2016. February 27, 2023. <https://www.udir.no/tall-og-forskning/finn-forskning/rapporter/nasjonale-prover-2013---fagmiljoenes-analyse-av-provene/>

4. Eurostat. Statistics Explained. Gloassary: Equivalised disposable income. Accessed August 12, 2022. <https://ec.europa.eu/eurostat/statistics-explained/index.php?title=Glossary:Equivalised_disposable_income>

5. Kendler KS, Neale MC, Kessler RC, et al. The lifetime history of major depression in women: Reliability of diagnosis and heritability. *Arch Gen Psychiatry*. 1993;50(11):863-870. doi:10.1001/archpsyc.1993.01820230054003

6. Strand BH, Dalgard OS, Tambs K, et al. Measuring the mental health status of the Norwegian population: A comparison of the instruments SCL-25, SCL-10, SCL-5 and MHI-5 (SF-36). *Nord J Psychiatry*. 2003;57(2):113-118. doi:10.1080/08039480310000932

7. Tambs K, Moum T. How well can a few questionnaire items indicate anxiety and depression? *Acta Psychiatrica Scandinavica*. 1993;87(5):364-367. doi:doi:10.1111/j.1600-0447.1993.tb03388.x

8. Fink P, Ørnbøl E, Huyse FJ, et al. A brief diagnostic screening instrument for mental disturbances in general medical wards. *Journal of Psychosomatic Research*. 2004/07/01/ 2004;57(1):17-24.

**Table S1. Test scores among children in MoBa and all children in Norway who**

**took the test, according to subject and test year.**

|  | **Test year** | **General population (N=465 908)** | | **MoBa population (N=94 043)** | |
| --- | --- | --- | --- | --- | --- |
|  |  | **N** | **Mean raw score (SD)** | **N** | **Mean raw score (SD)** |
| **Numeracy** | 2011 | 55 122 | 26.0 (8.5) | 14 | 30.1 (6.5) |
|  | 2012 | 54 790 | 26.7 (8.6) | 7510 | 27.7 (8.4) |
|  | 2013 | 56 688 | 25.1 (9.6) | 11 095 | 26.4 (9.2) |
|  | 2014 | 57 997 | 23.8 (9.7) | 12 037 | 25.3 (9.4) |
|  | 2015 | 58 032 | 23.4 (9.9) | 13 977 | 25.3 (9.8) |
|  | 2016 | 59 657 | 22.9 (9.6) | 15 640 | 25.3 (9.3) |
|  | 2017 | 58 710 | 22.8 (9.5) | 14 246 | 25.2 (9.3) |
|  | 2018 | 60 478 | 24.8 (9.5) | 11 897 | 27.6 (9.1) |
|  | Overall | 461 474 | 24.4 (9.5) | 86 416 | 25.9 (9.3) |
|  | Missing | 4434 | - | 7627 | - |
| **Literacy** | 2011 | 54 826 | 21.3 (5.9) | 14 | 24.0 (3.4) |
|  | 2012 | 54 319 | 18.6 (6.2) | 7434 | 19.3 (6.0) |
|  | 2013 | 56 254 | 21.2 (7.2) | 10 974 | 22.2 (6.8) |
|  | 2014 | 57 851 | 18.1 (6.9) | 12 023 | 19.2 (6.5) |
|  | 2015 | 57 906 | 19.5 (7.5) | 13 962 | 20.8 (7.3) |
|  | 2016 | 59 533 | 18.0 (7.5) | 15 626 | 19.9 (7.3) |
|  | 2017 | 58 192 | 19.0 (6.4) | 14 197 | 20.7 (6.1) |
|  | 2018 | 59 792 | 17.4 (6.1) | 11 822 | 19.1 (5.9) |
|  | Overall | 458 673 | 19.1 (6.9) | 86 052 | 20.2 (6.7) |
|  | Missing | 7235 | - | 7991 | - |

Abbrevation: MoBa, the Norwegian Mother, Father and Child Cohort.

Table S2. Description of the model specifications for the different inverse probability weights (IPWs).

| **Description of stabilized IPWs for missing data on covariates** | |  |
| --- | --- | --- |
| **Name** | **Model** |  |
| ipw_miss_c_early  Estimated among all individuals eligible for the early pregnancy trial. | Numerator:  Probability of being a complete case in the early pregnancy trial.  Denominator:  Probability of being a complete case in the early pregnancy trial given A_pp_,  L_pp,_ variables in Z with complete data (maternal age, parity, sleeping problems, anxiety pre-pregnancy, depression pre-pregnancy, chronic disease pre-pregnancy (in 5 groups^a^)). |  |
| ipw_miss_c_mid  Estimated among all individuals eligible for the early pregnancy trial, who additionally completed Q3. | Numerator:  Probability of being a complete case in the mid pregnancy trial.  Denominator:  Probability of being a complete case in the mid pregnancy trial given A_pp_, A_early_, L_pp_, variables in L_early_ with complete data (comedication use in early pregnancy),and variables in Z with complete data. |  |
| ipw_miss_c_late  Estimated among all individuals eligible for the early pregnancy trial, who additionally completed Q3 and Q4. | Numerator:  Probability of being a complete case in the late pregnancy trial.  Denominator:  Probability of being a complete case at in the late pregnancy trial given A_pp_, A_early_, A_mid_, L_pp_, variables in L_early_ and L_mid_ with complete data (comedication use in early and mid pregnancy^b^), and variables in Z with complete data. |  |
| **Description of stabilized IPWs for missing outcome data** | |  |
| **Name** | **Model** | |
| ipw_miss_o  Estimated among all individuals eligible for the early pregnancy trial. | Numerator:  Probability of having complete outcome data Y (separately for numeracy and literacy).  Denominator:  Probability of having outcome data given A_early_, Z (Maternal age, marital status, parity, BMI, education, smoking, alcohol, family income, LTH of MD, sleeping problems, anxiety pre-pregnancy, depression pre-pregnancy, chronic disease pre-pregnancy), L_early_ and birth month.  Missing data on covariates were adjusted for by IPW for missing data (ipw_miss_c_early). | |
| **Description of stabilized IPTWs** | | |
| **Name** | **Model** | |
| iptw_early  Estimated among all individuals eligible for the early pregnancy trial. | Numerator:  Probability of BZD and/or z-hypnotic use in early pregnancy.  Denominator:  Probability of BZD and/or z-hypnotic use in early pregnancy given  L_pp_ (comedication use during the 6 months prior to pregnancy), A_pp_ (BZD and/or z-hypnotic use during the 6 months prior to pregnancy),  Z (Maternal age, marital status, parity, BMI, education, smoking, alcohol, family income, LTH of MD, sleeping problems, anxiety pre-pregnancy, depression pre-pregnancy, chronic disease pre-pregnancy).  Missing data on covariates adjusted for by IPW for missing data (ipw_miss_c_early). | |
| iptw_mid  Estimated among all individuals eligible for the mid pregnancy trial. | Numerator:  Probability of BZD and/or z-hypnotic use in mid pregnancy.  Denominator:  Probability BZD and/or z-hypnotic use in mid pregnancy given  L_early_ (symptoms of depression and anxiety in early pregnancy and comedication use in early pregnancy), L_pp_, A_pp,_  Z,  interactions between (smoking*chronic disease pre-pregnancy), (BMI*chronic disease pre-pregnancy), (BMI*age), (BMI*smoking), (alcohol*smoking), (anxiety pre-pregnancy *depression pre-pregnancy).  Missing data on covariates were adjusted for by IPW for missing data (ipw_miss_c_early * ipw_miss_c_mid). | |
| iptw_late  Estimated among all individuals eligible for the late pregnancy trial. | Numerator:  Probability of BZD and/or z-hypnotic use in late pregnancy.  Denominator:  Probability of BZD and/or z-hypnotic use in late pregnancy given  L_mid_ (symptoms of depression and anxiety in mid pregnancy and comedication use in mid pregnancy^b^), L_early_, L_pp_, A_pp,_  Z,  interactions between (sleeping problems*symptoms of depression and anxiety in early pregnancy), (sleeping problems*symptoms of depression and anxiety in mid pregnancy), (sleeping problems*alcohol).  Missing data on covariates adjusted for by IPW for missing data (ipw_miss_c_early * ipw_miss_c_mid * ipw_miss_c_late). | |
| **Description of stabilized IPCWs** | |  |
| **Name** | **Model** | |
| ipcw_mid  Estimated among all individuals eligible for the early pregnancy trial, with complete covariate data. | Numerator:  Probability of being uncensored in Q3  Denominator:  Probability of being uncensored in Q3 given  A_pp_, A_early_, L_pp_, L_early_, Z.  Missing data on covariates adjusted for by IPW for missing data (ipw_miss_c_early* ipw_miss_c_mid). | |
| ipcw_late  Estimated among all individuals eligible for the early pregnancy trial, with complete covariate data, who completed Q3. | Numerator:  Probability of being uncensored in Q4  Denominator:  Probability of being uncensored in Q4 given  A_pp_, A_early_, A_mid_, L_pp_, L_early_, L_mid_^b^, Z.  Missing data on covariates adjusted for by IPW for missing data (ipw_miss_c_early * ipw_miss_c_mid * ipw_miss_c_late). | |

Abbreviations: IPCW, inverse probability of censoring weight; IPTW, inverse probability of treatment weight; IPW, inverse probability weight; MoBa, the Norwegian Mother, Father and Child Cohort Study; Q1, MoBa questionnaire 1; Q3, MoBa questionnaire 3; Q4, MoBa questionnaire 4.

^a^ Chronic disease pre-pregnancy was categorized in 5 groups: (1) asthma, (2) diabetes, (3) epilepsy, (4) hypertension, thyroid disorder, or other heart disease, (5) arthritis, lupus or Crohn’s disease.

^b^ Comedication use in mid pregnancy included only antidepressants, NSAIDs, paracetamol and opioids.

Table S3. Specification of the final combined stabilized weights used in each analysis.

| **For the analysis of the:** | **Final stabilized weight** |
| --- | --- |
| Early pregnancy trial | iptw_early * ipw_miss_c_early * ipw_miss_o |
| Mid pregnancy trial | iptw_mid * ipcw_mid *ipw_miss_o *  ipw_miss_c_mid * ipw_miss_c_early |
| Late pregnancy trial | iptw_late * ipcw_late * ipw_miss_o *  ipw_miss_c_late * ipw_miss_c_mid * ipw_miss_c_early |

Table S4. Use of benzodiazepines and z-hypnotics in the different emulated trials, No. (% of N).

|  | **Benzodiazepines** | | | **Z-hypnotics^d^** | **Any benzo-diazepine and/or z-hypnotic** |
| --- | --- | --- | --- | --- | --- |
|  | **Benzodiazepine-anxiolytics^a^** | **Benzo-diazepine-hypnotics^b^** | **Benzo-diazepine-antiepileptics^c^** |  |  |
| In the early pregnancy trial (N=7795) | 127 (1.63) | 12 (0.15) | 8 (0.10) | 77 (0.99) | 197 (2.53) |
| In the mid pregnancy trial (N=6685) | 21 (0.31) | 1 (0.01) | 1 (0.01) | 12 (0.18) | 34 (0.51) |
| In the late pregnancy trial (N=5743) | 13 (0.23) | 2 (0.03) | - | 10 (0.17) | 24 (0.42) |

^a^ N05BA (diazepam, oxazepam, alprazolam)

^b^ N05CD (nitrazepam, flunitrazepam, midazolam)

^c^ N03AE01 (clonazepam)

^d^ N05CF (zopiclone, zolpidem).

Table S5. Characteristics of individuals with incomplete data according to treatment initiation with benzodiazepines or z-hypnotics in each trial.

| **Characteristic** | **Individuals eligible for the early pregnancy trial (N=11 128), No. (%)** | | **Individuals eligible for the mid pregnancy trial (N=9764), No. (%)** | | **Individuals eligible for the late pregnancy trial (N=8491), No. (%)** | |
| --- | --- | --- | --- | --- | --- | --- |
|  | **Treatment initiation in early pregnancy** | **No treatment initiation in early pregnancy** | **Treatment initiation in mid pregnancy** | **No treatment initiation in mid pregnancy** | **Treatment initiation in late pregnancy** | **No treatment initiation in late pregnancy** |
| No. of participants | 273 (2.5) | 10 855 (97.5) | 56 (0.6) | 9708 (99.4) | 35 (0.4) | 8456 (99.6) |
| Age |  |  |  |  |  |  |
| <25 | 32 (11.7) | 1564 (14.4) | 5 (8.9) | 1348 (13.9) | 3 (8.6) | 1102 (13.0) |
| 25-29 | 72 (23.4) | 3347 (30.8) | 14 (25.0) | 3017 (31.1) | 9 (25.7) | 2667 (31.5) |
| 30-34 | 92 (33.7) | 3918 (36.1) | 28 (50.0) | 3527 (36.3) | 15 (42.9) | 3090 (36.6) |
| ≥35 | 77 (28.2) | 2026 (18.7) | 9 (16.1) | 1816 (18.7) | 8 (22.9) | 1597 (18.9) |
| Parity |  |  |  |  |  |  |
| Primiparous | 136 (49.8) | 5295 (48.8) | 19 (33.9) | 4804 (49.5) | 17 (48.6) | 4248 (50.2) |
| Multiparous | 137 (50.2) | 5560 (51.2) | 37 (66.1) | 4904 (50.5) | 18 (51.4) | 4208 (49.8) |
| Marital status |  |  |  |  |  |  |
| Married/cohabitant | 229 (83.9) | 10 055 (92.6) | 53 (94.6) | 9044 (93.2) | 32 (91.4) | 7908 (93.5) |
| Other | 39 (14.3) | 725 (6.7) | 2 (3.6) | 598 (6.2) | 3 (8.6) | 492 (5.8) |
| Missing | 5 (1.8) | 75 (0.7) | 1 (1.8) | 66 (0.7) |  | 56 (0.7) |
| Pre-pregnancy BMI |  |  |  |  |  |  |
| < 18.5 | 19 (7.0) | 421 (3.9) | 5 (8.9) | 359 (3.7) | 2 (5.7) | 296 (3.5) |
| 18.5-24.9 | 157 (57.5) | 6619 (61.0) | 30 (53.6) | 5957 (61.4) | 20 (57.1) | 5244 (62.0) |
| 25.0-29.9 | 63 (23.1) | 2280 (21.0) | 12 (21.4) | 2058 (21.2) | 8 (22.9) | 1806 (21.4) |
| ≥ 30 | 27 (9.9) | 1215 (11.1) | 8 (14.3) | 1071 (11.0) | 4 (11.4) | 895 (10.6) |
| Missing | 7 (2.5) | 320 (3.0) | 1 (1.8) | 263 (2.7) | 1 (2.9) | 215 (2.5) |
| Alcohol^a^ |  |  |  |  |  |  |
| No | 235 (86.1) | 9555 (88.0) | 47 (83.9) | 8592 (88.5) | 30 (85.7) | 7512 (88.8) |
| Yes | 14 (5.1) | 392 (3.6) | 3 (5.4) | 340 (3.5) | 1 (2.9) | 302 (3.6) |
| Missing | 24 (8.8) | 908 (8.4) | 6 (10.7) | 776 (8.0) | 4 (11.4) | 642 (7.6) |
| Smoking^b^ |  |  |  |  |  |  |
| No | 154 (56.4) | 7387 (68.1) | 35 (62.5) | 6693 (68.9) | 30 (85.7) | 5871 (69.4) |
| Yes | 80 (29.3) | 1531 (14.1) | 8 (14.3) | 1305 (13.4) | 2 (5.7) | 1095 (12.9) |
| Missing | 39 (14.3) | 1937 (17.8) | 13 (23.2) | 1710 (17.6) | 3 (8.6) | 1490 (17.6) |
| Educational level^c^ |  |  |  |  |  |  |
| 10-year primary school or less | 59 (21.6) | 1548 (14.3) | 9 (16.1) | 1276 (13.1) |  | 1006 (11.9) |
| Secondary/vocational school | 83 (30.4) | 3184 (29.3) | 14 (25.0) | 2873 (29.6) | 13 (37.1) | 2480 (29.3) |
| College or advanced degree | 125 (45.8) | 5928 (54.6) | 33 (58.9) | 5437 (56.0) | 22 (62.9) | 4879 (57.7) |
| Missing | 6 (2.2) | 195 (1.8) | - | 120 (1.2) | - . | 91 (1.1) |
| Family income, ITNR^d^ |  |  |  |  |  |  |
| <2 | 145 (53.1) | 4711 (43.4) | 22 (39.3) | 4157 (42.8) | 9 (25.7) | 3547 (41.9) |
| ≥2 | 123 (45.1) | 5938 (54.7) | 33 (58.9) | 5418 (55.8) | 26 (74.3) | 4803 (56.8) |
| Missing | 5 (1.8) | 206 (1.9) | 1 (1.8) | 133 (1.4) |  | 106 (1.3) |
| Anxiety pre-pregnancy | 170 (62.3) | 3245 (29.9) | 35 (62.5) | 2850 (29.4) | 18 (51.4) | 2439 (28.8) |
| Depression pre-pregnancy | 193 (70.7) | 6030 (55.6) | 37 (66.1) | 5375 (55.4) | 24 (68.6) | 4652 (55.0) |
| Sleeping problems in early pregnancy | 150 (54.9) | 3057 (28.2) | 19 (33.9) | 2679 (27.6) | 15 (42.9) | 2296 (27.2) |
| LTH of MD | 113 (41.4) | 5709 (52.6) | 26 (46.4) | 5112 (52.7) | 21 (60.0) | 4472 (52.9) |
| Missing | 4 (1.5) | 197 (1.8) | 1 (1.8) | 164 (1.7) |  | 132 (1.6) |
| Chronic disease pre-pregnancy^e^ | 88 (32.2) | 1988 (18.3) | 12 (21.4) | 1756 (18.1) | 7 (20.0) | 1506 (17.8) |
| Benzodiazepine and/or z-hypnotic use during the 6 months prior to pregnancy | 152 (55.7) | 307 (2.8) | 11 (19.6) | 264 (2.7) | 5 (14.3) | 226 (2.7) |
| Symptoms of depression/anxiety at gestational week 17^f^, mean (SD) | - | - | 1.0 (1.6) | 0.8 (1.5) | 1.6 (1.8) | 0.8 (1.5) |
| Missing | - | - | 2 (3.6) | 426 (4.4) | 3 (8.6) | 341 (4.0) |
| Symptoms of depression/anxiety at gestational week 30^f^, mean (SD) | - | - | - | - | 2.0 (2.2) | 0.7 (1.5) |
| Missing | - | - | - | - | 2 (5.7) | 226 (2.7) |
| Co-medication use during the 6 months prior to pregnancy |  |  |  |  |  |  |
| NSAIDs | 45 (16.5) | 1374 (12.7) | 5 (8.9) | 1266 (13.0) | 6 (17.1) | 1123 (13.3) |
| Opioids | 22 (8.1) | 259 (2.4) | 3 (5.4) | 228 (2.3) | 1 (2.9) | 203 (2.4) |
| Paracetamol | 83 (30.4) | 3224 (29.7) | 16 (28.6) | 2932 (30.2) | 11 (31.4) | 2579 (30.5) |
| Antidepressants | 90 (33.0) | 1267 (11.7) | 12 (21.4) | 1134 (11.7) | 8 (22.9) | 979 (11.6) |
| Antipsychotics | 19 (7.0) | 85 (0.8) | 1 (1.8) | 75 (0.8) | - | 64 (0.8) |
| Antiepileptics^g^ | 11 (4.0) | 75 (0.7) |  | 63 (0.6) |  | 51 (0.6) |
| Co-medication use in early pregnancy |  |  |  |  |  |  |
| NSAIDs | - | - | 7 (12.5) | 640 (6.6) | 1 (2.9) | 564 (6.7) |
| Opioids | - | - | 6 (10.7) | 181 (1.9) | - | 160 (1.9) |
| Paracetamol | - | - | 35 (62.5) | 4096 (42.2) | 16 (45.7) | 3551 (42.0) |
| Antidepressants | - | - | 14 (25.0) | 760 (7.8) | 4 (11.4) | 652 (7.7) |
| Antipsychotics | - | - | 3 (5.4) | 128 (1.3) | 1 (2.9) | 110 (1.3) |
| Antiepileptics^g^ | - | - | - | 61 (0.6) |  | 50 (0.6) |
| Co-medication use in mid pregnancy |  |  |  |  |  |  |
| NSAIDs | - | - | - | - | 1 (2.9) | 126 (1.5) |
| Opioids | - | - | - | - | 2 (5.7) | 105 (1.2) |
| Paracetamol | - | - | - | - | 10 (28.6) | 2360 (27.9) |
| Antidepressants | - | - | - | - | 5 (14.3) | 242 (2.9) |
| Antipsychotics | - | - | - | - | 1 (2.9) | 42 (0.5) |
| Antiepileptics^g^ | - | - | - | - | - | 13 (0.2) |

Abbreviations: BMI, body mass index; ITNR, income to needs ratio; LTH of MD, Life Time History of Major Depression; NSAIDs, nonsteroidal anti-inflammatory drugs.

^a^ Alcohol use was measured in MoBa questionnaire 1.

^b^ Smoking was measured in early pregnancy.

^c^ Educational level assessed in the child’s birth year.

^d^ Family income was assessed by ITNR

^e^ Chronic disease included asthma, diabetes, hypertension, epilepsy, arthritis, other heart disease, thyroid disorder, lupus or Crohn’s disease, reported before pregnancy.

^f^ Presence of symptoms of depression or anxiety indicated on the 5-item short version of the Hopkins Symptoms Checklist (SCL-5)

^g^ Antiepileptic use does not include clonazepam (N03AE01).

Table S6. Overview of the gestational ages at birth for the eligible individuals in each trial, No. (% of N).

| **Gestational age at birth** | **Eligible for the early pregnancy trial (N=7795)** | **Eligible for the mid pregnancy trial (N=6685)** | **Eligible for the late pregnancy trial (N=5743)** |
| --- | --- | --- | --- |
| 17 – 28 weeks | 26 (0.3) | 11 (0.2) | 7 (0.1) |
| ≥ 29 weeks | 7739 (99.3) | 6650 (99.5) | 5715 (99.5) |
| Missing | 30 (0.4) | 24 (0.3) | 21 (0.4) |

**Table S7. National test results of the MoBa children presented as z scores, for the children of the eligible participants in each trial.**

|  | **Literacy** | | | **Numeracy** | | |
| --- | --- | --- | --- | --- | --- | --- |
|  | **N** | **Mean score (SD)** | **Median** **scores (P25, P75)** | **N** | **Mean score (SD)** | **Median** **scores (P25, P75)** |
| **MoBa population (N=94 043)** | 84 948 | 0.21 (0.97) | 0.37 (-0.48, 0.97) | 85 980 | 0.20 (0.98) | 0.23 (-0.54, 0.96) |
| **Eligible for the early pregnancy trial (N=7795)** | 7012 | 0.21 (0.98) | 0.37 (-0.48, 1.02) | 7112 | 0.15 (1.00) | 0.19 (-0.61, 0.95) |
| **Eligible for the mid pregnancy trial (N=6685)** | 6043 | 0.23 (0.97) | 0.37 (-0.47, 1.02) | 6123 | 0.17 (0.99) | 0.21 (-0.57, 0.96) |
| **Eligible for the late pregnancy trial (N=5743)** | 5233 | 0.25 (0.96) | 0.40 (-0.46, 1.03) | 5304 | 0.19 (0.98) | 0.23 (-0.55, 0.96) |

Abbrevations: MoBa, The Norwegian Mother, Father and Child cohort

P25 indicate the 25th percentile, and P75 indicate the 75th percentile of the z-scores.

**Table S8. Overview of the number of children with missing information on the outcomes in each trial, No. (% of N).**

| **Missing information on test scores in** | **Eligible for the early pregnancy trial (N=7795)** | **Eligible for the mid pregnancy trial (N=6685)** | **Eligible for the late pregnancy trial (N=5743)** |
| --- | --- | --- | --- |
| **Literacy** | 783 (10.0) | 642 (9.6) | 510 (8.9) |
| **Numeracy** | 683 (8.8) | 562 (8.4) | 439 (7.6) |

Table S9. Characteristics of individuals in each trial according to whether outcome information was available for the numeracy test.

| **Characteristic** | **Individuals eligible for the early pregnancy trial (N=7795), No. (%)** | | **Individuals eligible for the mid pregnancy trial (N=6685), No. (%)** | | **Individuals eligible for the late pregnancy trial (N=5743), No. (%)** | |
| --- | --- | --- | --- | --- | --- | --- |
|  | **Outcome information** | **Missing outcome information** | **Outcome information** | **Missing outcome information** | **Outcome information** | **Missing outcome information** |
| No. of participants | 7112 | 683 | 6123 | 562 | 5304 | 439 |
| Initiate treatment in the trial | 178 (2.5) | 19 (2.8) | 31 (0.5) | 3 (0.5) | 23 (0.4) | 1 (0.2) |
| Age |  |  |  |  |  |  |
| <25 | 1033 (14.5) | 103 (15.1) | 861 (14.1) | 83 (14.8) | 699 (13.2) | 59 (13.4) |
| 25-29 | 2196 (30.9) | 215 (31.5) | 1921 (31.4) | 174 (31.0) | 1697 (32.0) | 142 (32.3) |
| 30-34 | 2552 (35.9) | 227 (33.2) | 2205 (36.0) | 195 (34.7) | 1916 (36.1) | 150 (34.2) |
| ≥35 | 1331 (18.7) | 138 (20.2) | 1136 (18.5) | 110 (19.6) | 992 (18.7) | 88 (20.0) |
| Parity |  |  |  |  |  |  |
| Primiparous | 3363 (47.3) | 345 (50.5) | 2932 (47.9) | 295 (47.5) | 2589 (48.8) | 231 (52.6) |
| Multiparous | 3749 (52.7) | 338 (49.5) | 3191 (52.1) | 267 (52.5) | 2715 (51.2) | 208 (47.4) |
| Marital status |  |  |  |  |  |  |
| Married/cohabitant | 6646 (93.4) | 630 (92.2) | 5769 (94.2) | 521 (92.7) | 5003 (94.3) | 408 (92.9) |
| Other | 466 (6.6) | 53 (7.8) | 354 (5.8) | 41 (7.3) | 301 (5.7) | 31 (7.1) |
| Pre-pregnancy BMI |  |  |  |  |  |  |
| < 18.5 | 274 (3.9) | 33 (4.8) | 223 (3.6) | 28 (5.0) | 184 (3.5) | 18 (4.1) |
| 18.5-24.9 | 4422 (62.2) | 403 (59.0) | 3805 (62.1) | 331 (58.9) | 3312 (62.4) | 266 (60.6) |
| 25.0-29.9 | 1589 (22.3) | 131 (19.2) | 1386 (22.6) | 109 (19.4) | 1204 (22.7) | 86 (19.6) |
| ≥ 30 | 827 (11.6) | 116 (17.0) | 709 (11.6) | 94 (16.7) | 604 (11.4) | 69 (15.7) |
| Alcohol^a^ | 272 (3.8) | 18 (2.6) | 221 (3.6) | 13 (2.3) | 189 (3.6) | 11 (2.5) |
| Smoking^b^ | 1215 (17.1) | 107 (15.7) | 974 (15.9) | 80 (14.2) | 810 (15.3) | 57 (13.0) |
| Educational level^c^ |  |  |  |  |  |  |
| 10-year primary school or less | 964 (13.6) | 129 (18.9) | 752 (12.3) | 101 (18.0) | 580 (10.9) | 67 (15.3) |
| Secondary/vocational school | 2204 (31.0) | 190 (27.8) | 1900 (31.0) | 156 (27.7) | 1611 (30.4) | 124 (28.2) |
| College or advanced degree | 3944 (55.5) | 364 (53.3) | 3471 (56.7) | 305 (54.3) | 3113 (58.7) | 248 (56.5) |
| Family income, ITNR^d^ |  |  |  |  |  |  |
| <2 | 3136 (44.1) | 333 (48.8) | 2639 (43.1) | 269 (47.9) | 2240 (42.2) | 201 (45.8) |
| ≥2 | 3976 (55.9) | 350 (51.2) | 3484 (56.9) | 293 (52.1) | 3064 (57.8) | 238 (54.2) |
| Anxiety pre-pregnancy | 2119 (29.8) | 206 (30.2) | 1767 (28.9) | 160 (28.5) | 1303 (28.3) | 122 (27.8) |
| Depression pre-pregnancy | 3957 (55.6) | 380 (55.6) | 3372 (55.1) | 314 (55.9) | 2915 (55.0) | 231 (52.6) |
| Sleeping problems in early pregnancy | 2009 (28.2) | 216 (31.6) | 1647 (26.9) | 172 (30.6) | 1407 (26.5) | 131 (29.8) |
| LTH of MD | 3772 (53.0) | 387 (56.7) | 3241 (52.9) | 324 (57.7) | 2821 (53.2) | 261 (59.5) |
| Chronic disease pre-pregnancy^e^ | 1297 (18.2) | 146 (21.4) | 1082 (17.7) | 119 (21.2) | 922 (17.4) | 86 (19.6) |
| Benzodiazepine and/or z-hypnotic use during the 6 months prior to pregnancy | 278 (3.9) | 38 (5.6) | 163 (2.7) | 24 (4.3) | 134 (3.5) | 19 (4.3) |
| Symptoms of depression/anxiety at gestational week 17^f^, mean (SD) | - | - | 0 (1) | 0.02 (0.9) | 0 (1) | 0 (0.9) |
| Symptoms of depression/anxiety at gestational week 30^f^, mean (SD) | - | - | - | - | 0 (1) | 0.01 (1) |
| Co-medication use during the 6 months prior to pregnancy |  |  |  |  |  |  |
| NSAIDs | 908 (12.8) | 89 (13.0) | 801 (13.1) | 76 (13.5) | 704 (13.3) | 62 (14.1) |
| Opioids | 182 (2.6) | 15 (2.2) | 150 (2.4) | 12 (2.1) | 130 (2.5) | 8 (1.8) |
| Paracetamol | 2118 (29.8) | 223 (32.7) | 1853 (30.3) | 180 (32.0) | 1621 (30.6) | 138 (31.4) |
| Antidepressants | 880 (12.4) | 98 (14.3) | 725 (11.8) | 78 (13.9) | 624 (11.8) | 60 (13.7) |
| Antipsychotics | 68 (1.0) | 6 (0.9) | 48 (0.8) | 5 (0.9) | 40 (0.8) | 5 (1.1) |
| Antiepileptics^g^ | 54 (0.8) | 10 (1.5) | 43 (0.7) | 5 (0.9) | 35 (0.7) | 2 (0.5) |
| Co-medication use in early pregnancy |  |  |  |  |  |  |
| NSAIDs | - | - | 424 (6.9) | 39 (6.9) | 367 (6.9) | 30 (6.8) |
| Opioids | - | - | 108 (1.8) | 14 (2.5) | 91 (1.7) | 8 (1.8) |
| Paracetamol | - | - | 2605 (42.5) | 249 (44.3) | 2247 (42.4) | 192 (43.7) |
| Antidepressants | - | - | 493 (8.1) | 49 (8.7) | 418 (7.9) | 34 (7.7) |
| Antipsychotics | - | - | 84 (1.4) | 5 (0.9) | 67 (1.3) | 5 (1.1) |
| Antiepileptics^g^ | - | - | 40 (0.7) | 5 (0.9) | 34 (0.6) | 2 (0.5) |
| Co-medication use in mid pregnancy |  |  | - | - |  |  |
| NSAIDs | - | - | - | - | 82 (1.5) | 7 (1.6) |
| Opioids | - | - | - | - | 64 (1.2) | 7 (1.6) |
| Paracetamol | - | - | - | - | 1456 (27.5) | 132 (30.1) |
| Antidepressants | - | - | - | - | 152 (2.9) | 16 (3.6) |
| Antipsychotics | - | - | - | - | 23 (0.4) | - |
| Antiepileptics^g^ | - | - | - | - | 6 (0.1) | - |

Abbreviations: BMI, body mass index; ITNR, income to needs ratio; LTH of MD, Life Time History of Major Depression; NSAIDs, nonsteroidal anti-inflammatory drugs.

^a^ Alcohol use was measured in MoBa questionnaire 1.

^b^ Smoking was measured in early pregnancy.

^c^ Educational level assessed in the child’s birth year.

^d^ Family income was assessed by ITNR

^e^ Chronic disease included asthma, diabetes, hypertension, epilepsy, arthritis, other heart disease, thyroid disorder, lupus or Crohn’s disease, reported before pregnancy.

^f^ Presence of symptoms of depression or anxiety indicated on the 5-item short version of the Hopkins Symptoms Checklist (SCL-5)

^g^ Antiepileptic use does not include clonazepam (N03AE01).

Table S10. Characteristics of individuals in each trial according to whether outcome information is available for the literacy test.

| **Characteristic** | **Individuals eligible for the early pregnancy trial (N=7795), No. (%)** | | **Individuals eligible for the mid pregnancy trial (N=6685), No. (%)** | | **Individuals eligible for the late pregnancy trial (N=5743), No. (%)** | |
| --- | --- | --- | --- | --- | --- | --- |
|  | **Outcome information** | **Missing outcome information** | **Outcome information** | **Missing outcome information** | **Outcome information** | **Missing outcome information** |
| No. of participants | 7012 | 783 | 6043 | 642 | 5233 | 510 |
| Initiate treatment in the trial | 177 (2.5) | 20 (2.6) | 31 (0.5) | 3 (0.5) | 23 (0.4) | 1 (0.2) |
| Age |  |  |  |  |  |  |
| <25 | 1018 (14.5) | 118 (15.1) | 848 (14.0) | 96 (15.0) | 688 (13.1) | 70 (13.7) |
| 25-29 | 2166 (30.9) | 245 (31.3) | 1896 (31.4) | 199 (31.0) | 1678 (32.1) | 161 (31.6) |
| 30-34 | 2526 (36.0) | 253 (32.3) | 2187 (36.2) | 213 (33.2) | 1894 (36.2) | 172 (33.7) |
| ≥35 | 1302 (18.6) | 167 (21.3) | 1112 (18.4) | 134 (20.9) | 973 (18.6) | 107 (21.0) |
| Parity |  |  |  |  |  |  |
| Primiparous | 3322 (47.4) | 386 (49.3) | 2897 (47.9) | 330 (51.4) | 2553 (48.8) | 267 (52.4) |
| Multiparous | 3690 (52.6) | 397 (50.7) | 3146 (52.1) | 312 (48.6) | 2680 (51.2) | 243 (47.6) |
| Marital status |  |  |  |  |  |  |
| Married/cohabitant | 6552 (93.4) | 724 (92.5) | 5695 (94.2) | 595 (92.7) | 4938 (94.4) | 473 (92.7) |
| Other | 460 (6.6) | 59 (7.5) | 348 (5.8) | 47 (7.3) | 295 (5.6) | 37 (7.3) |
| Pre-pregnancy BMI |  |  |  |  |  |  |
| < 18.5 | 276 (3.9) | 31 (4.0) | 226 (3.7) | 25 (3.9) | 186 (3.5) | 16 (3.1) |
| 18.5-24.9 | 4364 (62.2) | 461 (58.9) | 3762 (62.3) | 374 (58.3) | 3272 (62.5) | 306 (60.0) |
| 25.0-29.9 | 1563 (22.3) | 157 (20.1) | 1363 (22.6) | 132 (20.6) | 1186 (22.7) | 104 (20.4) |
| ≥ 30 | 809 (11.5) | 134 (17.1) | 692 (11.5) | 111 (17.3) | 589 (11.3) | 84 (16.5) |
| Alcohol^a^ | 270 (3.9) | 20 (2.6) | 220 (3.6) | 14 (2.2) | 188 (3.6) | 12 (2.4) |
| Smoking^b^ | 1195 (17.0) | 127 (16.2) | 958 (15.9) | 96 (15.0) | 793 (15.2) | 74 (14.5) |
| Educational level^c^ |  |  |  |  |  |  |
| 10-year primary school or less | 948 (13.5) | 145 (18.5) | 738 (12.2) | 115 (17.9) | 569 (10.9) | 78 (15.3) |
| Secondary/vocational school | 2158 (30.8) | 236 (30.1) | 1867 (30.9) | 189 (29.4) | 1583 (30.3) | 152 (29.8) |
| College or advanced degree | 3906 (55.7) | 402 (51.3) | 3438 (56.9) | 338 (52.6) | 3081 (58.9) | 280 (54.9) |
| Family income, ITNR^d^ |  |  |  |  |  |  |
| <2 | 3089 (44.1) | 380 (48.5) | 2605 (43.1) | 303 (47.2) | 2212 (42.3) | 229 (44.9) |
| ≥2 | 3923 (55.9) | 403 (51.5) | 3438 (56.9) | 339 (52.8) | 3021 (57.7) | 281 (55.1) |
| Anxiety pre-pregnancy | 2088 (29.8) | 237 (30.3) | 1744 (28.9) | 183 (28.5) | 1482 (28.3) | 143 (28.0) |
| Depression pre-pregnancy | 3890 (55.5) | 447 (57.1) | 3318 (54.9) | 368 (57.3) | 2862 (54.7) | 284 (55.7) |
| Sleeping problems in early pregnancy | 1985 (28.3) | 240 (30.7) | 1630 (27.0) | 189 (29.4) | 1390 (26.6) | 148 (29.0) |
| LTH of MD | 3728 (53.2) | 431 (55.0) | 3208 (53.1) | 357 (55.6) | 2793 (53.4) | 289 (56.7) |
| Chronic disease pre-pregnancy^e^ | 1279 (18.2) | 164 (20.9) | 1065 (17.6) | 136 (21.2) | 907 (17.3) | 101 (19.8) |
| Benzodiazepine and/or z-hypnotic use during the 6 months prior to pregnancy | 272 (3.9) | 44 (5.6) | 159 (2.6) | 28 (4.4) | 130 (2.5) | 23 (4.5) |
| Symptoms of depression/anxiety at gestational week 17^f^, mean (SD) | - | - | 0 (1) | 0 (0.9) | 0 (1) | -0.02 (0.9) |
| Symptoms of depression/anxiety at gestational week 30^f^, mean (SD) | - | - | - | - | 0 (1) | 0 (1) |
| Co-medication use during the 6 months prior to pregnancy |  |  |  |  |  |  |
| NSAIDs | 900 (12.8) | 97 (12.4) | 793 (13.1) | 84 (13.1) | 700 (13.4) | 66 (12.9) |
| Opioids | 179 (2.6) | 18 (2.3) | 148 (2.4) | 14 (2.2) | 129 (2.5) | 9 (1.8) |
| Paracetamol | 2088 (29.8) | 253 (32.3) | 1827 (30.2) | 206 (32.1) | 1601 (30.6) | 158 (31.0) |
| Antidepressants | 872 (12.4) | 106 (13.5) | 721 (11.9) | 82 (12.8) | 619 (11.8) | 65 (12.7) |
| Antipsychotics | 66 (0.9) | 8 (1.0) | 47 (0.8) | 6 (0.9) | 39 (0.7) | 6 (1.2) |
| Antiepileptics^g^ | 56 (0.8) | 8 (1.0) | 45 (0.7) | 3 (0.5) | 36 (0.7) | 1 (0.2) |
| Co-medication use in early pregnancy |  |  |  |  |  |  |
| NSAIDs | - | - | 421 (7.0) | 42 (6.5) | 366 (7.0) | 31 (6.1) |
| Opioids | - | - | 108 (1.8) | 14 (2.2) | 90 (1.7) | 9 (1.8) |
| Paracetamol | - | - | 2568 (42.5) | 286 (44.5) | 2216 (42.3) | 223 (43.7) |
| Antidepressants | - | - | 490 (8.1) | 52 (8.1) | 414 (7.9) | 38 (7.5) |
| Antipsychotics | - | - | 81 (1.3) | 8 (1.2) | 64 (1.2) | 8 (1.6) |
| Antiepileptics^g^ | - | - | 41 (0.7) | 4 (0.6) | 34 (0.6) | 2 (0.4) |
| Co-medication use in mid pregnancy |  |  | - | - |  |  |
| NSAIDs | - | - | - | - | 81 (1.5) | 8 (1.6) |
| Opioids | - | - | - | - | 61 (1.2) | 10 (2.0) |
| Paracetamol | - | - | - | - | 1437 (27.5) | 151 (29.6) |
| Antidepressants | - | - | - | - | 147 (2.8) | 21 (4.1) |
| Antipsychotics | - | - | - | - | 23 (0.4) | - |
| Antiepileptics^g^ | - | - |  |  | 6 (0.1) | - |

Abbreviations: BMI, body mass index; ITNR, income to needs ratio; LTH of MD, Life Time History of Major Depression; NSAIDs, nonsteroidal anti-inflammatory drugs.

^a^ Alcohol use was measured in MoBa questionnaire 1.

^b^ Smoking was measured in early pregnancy.

^c^ Educational level assessed in the child’s birth year.

^d^ Family income was assessed by ITNR

^e^ Chronic disease included asthma, diabetes, hypertension, epilepsy, arthritis, other heart disease, thyroid disorder, lupus or Crohn’s disease, reported before pregnancy.

^f^ Presence of symptoms of depression or anxiety indicated on the 5-item short version of the Hopkins Symptoms Checklist (SCL-5)

^g^ Antiepileptic use does not include clonazepam (N03AE01).

**Table S11. Characteristics of the final combined stabilized IPWs.**

|  | **Literacy** | | **Numeracy** | |
| --- | --- | --- | --- | --- |
|  | **Mean (SD)** | **Min – max** | **Mean (SD)** | **Min – max** |
| **Eligible for the early pregnancy trial** |  |  |  |  |
| Treatment initiation in the early pregnancy trial | 0.76 (1.10) | 0.03 – 8.48 | 0.78 (1.15) | 0.02 – 8.78 |
| No treatment initiation in the early pregnancy trial | 1.00 (0.20) | 0.31 – 6.03 | 1.00 (0.22) | 0.20 – 5.89 |
| **Eligible for the mid pregnancy trial** |  |  |  |  |
| Treatment initiation in the mid pregnancy trial | 0.54 (0.89) | 0.01 – 3.51 | 0.53 (0.88) | 0.01 – 3.47 |
| No treatment initiation in the mid pregnancy trial | 0.98 (0.16) | 0.33 – 2.89 | 0.98 (0.18) | 0.21 – 3.97 |
| **Eligible for the late pregnancy trial** |  |  |  |  |
| Treatment initiation in the late pregnancy trial | 0.65 (0.98) | 0.01 – 4.23 | 0.71 (1.02) | 0.01 – 4.21 |
| No treatment initiation in the late pregnancy trial | 0.90 (0.17) | 0.32 – 3.58 | 0.90 (0.19) | 0.29 – 3.67 |

Abbreviations: IPW, inverse probability weight; max, maximum; min, minimum; SD, standard deviation.

**Table S12. Characteristics of the stabilized IPTWs.**

|  | **Literacy** | | **Numeracy** | |
| --- | --- | --- | --- | --- |
|  | **Mean (SD)** | **Min – max** | **Mean (SD)** | **Min – max** |
| **Eligible for the early pregnancy trial** |  |  |  |  |
| Treatment initiation in the early pregnancy trial | 0.76 (1.10) | 0.03 – 8.01 | 0.76 (1.10) | 0.03 – 8.01 |
| No treatment initiation in the early pregnancy trial | 1.00 (0.13) | 0.98 – 6.42 | 1.00 (0.13) | 0.98 – 6.42 |
| **Eligible for the mid pregnancy trial** |  |  |  |  |
| Treatment initiation in the mid pregnancy trial | 0.57 (0.92) | 0.01 – 3.60 | 0.57 (0.92) | 0.01 – 3.60 |
| No treatment initiation in the mid pregnancy trial | 1.00 (0.02) | 0.99 – 1.91 | 1.00 (0.02) | 0.99 – 1.91 |
| **Eligible for the late pregnancy trial** |  |  |  |  |
| Treatment initiation in the late pregnancy trial | 0.78 (1.24) | 0.01 – 5.34 | 0.78 (1.24) | 0.01 – 5.34 |
| No treatment initiation in the late pregnancy trial | 1.00 (0.04) | 1.00 – 3.83 | 1.00 (0.04) | 1.00 – 3.83 |

Abbreviations: IPTW, inverse probability of treatment weight; max, maximum; min, minimum; SD, standard deviation.

**Table S13. Results from sensitivity analysis, changing the eligibility criteria in each trial to include only individuals who did not use benzodiazepines and/or z-hypnotics during the 6 months prior to pregnancy.** Crude and weighted mean differences in the children’s test scores in literacy and numeracy according to the mothers’ initiation of benzodiazepines and/or z-hypnotics in each trial (early pregnancy trial N=7479, mid pregnancy trial N =6498, late pregnancy trial N=5590)

|  | **N** | **Mean z-score (SD)** | **Crude mean difference (95% CI)** | **Weighted mean difference (95% CI)** |
| --- | --- | --- | --- | --- |
| **Literacy** |  |  |  |  |
| Treatment initiation in the early pregnancy trial | 95 | 0.05 (1.0) | -0.16 (-0.37, 0.04) | 0.05 (-0.20, 0.29) |
| No treatment initiation in the early pregnancy trial | 7384 | 0.21 (1.0) |  |  |
| Treatment initiation in the mid pregnancy trial | 27 | 0.09 (0.9) | -0.13 (-0.47, 0.25) | -0.17 (-0.73, 1.31) |
| No treatment initiation in the mid pregnancy trial | 6471 | 0.23 (1.0) |  |  |
| Treatment initiation in the late pregnancy trial | 20 | -0.03 (1.1) | -0.27 (-0.83, 0.16) | -0.31 (-1.84, 0.49) |
| No treatment initiation in the late pregnancy trial | 5570 | 0.24 (1.0) |  |  |
| **Numeracy** |  |  |  |  |
| Treatment initiation in the early pregnancy trial | 95 | 0.02 (1.1) | -0.12 (-0.34, 0.11) | 0 (-0.32, 0.38) |
| No treatment initiation in the early pregnancy trial | 7384 | 0.14 (1.0) |  |  |
| Treatment initiation in the mid pregnancy trial | 27 | -0.09 (1.0) | -0.25 (-0.61, 0.15) | -0.09 (-0.59, 1.44) |
| No treatment initiation in the mid pregnancy trial | 6471 | 0.16 (1.0) |  |  |
| Treatment initiation in the late pregnancy trial | 20 | 0.06 (1.1) | -0.12 (-0.63, 0.40) | 0.46 (-0.48, 1.44) |
| No treatment initiation in the late pregnancy trial | 5570 | 0.18 (1.0) |  |  |

Table S14. Characteristics of individuals with complete data according to treatment initiation with benzodiazepines or z-hypnotics, for the trials in the sensitivity analysis not restricting to individuals with a history of anxiety and/or depression.

| **Characteristic** | **Individuals eligible for the early pregnancy trial (N=67 280), No. (%)** | | **Individuals eligible for the mid pregnancy trial (N=60 820), No. (%)** | | **Individuals eligible for the late pregnancy trial (N=54 400), No. (%)** | |
| --- | --- | --- | --- | --- | --- | --- |
|  | **Treatment initiation in early pregnancy** | **No treatment initiation in early pregnancy** | **Treatment initiation in mid pregnancy** | **No treatment initiation in mid pregnancy** | **Treatment initiation in late pregnancy** | **No treatment initiation in late pregnancy** |
| No. of participants | 370 (0.5) | 66 910 (99.5) | 94 (0.2) | 60 726 (99.8) | 74 (0.1) | 54 326 (99.9) |
| Age |  |  |  |  |  |  |
| <25 | 40 (10.8) | 7373 (11.0) | 9 (9.6) | 6509 (10.7) | 5 (6.8) | 5532 (10.2) |
| 25-29 | 87 (23.5) | 22 402 (33.5) | 24 (25.5) | 20 490 (33.8) | 15 (20.3) | 18 479 (34.0) |
| 30-34 | 138 (37.3) | 25 689 (38.4) | 44 (46.8) | 23 404 (38.5) | 36 (48.6) | 21 075 (38.8) |
| ≥35 | 105 (28.4) | 11 446 (17.1) | 17 (18.1) | 10 323 (17.0) | 18 (24.3) | 9240 (17.0) |
| Parity |  |  |  |  |  |  |
| Primiparous | 176 (47.6) | 29 608 (44.3) | 31 (33.0) | 27 198 (44.8) | 31 (41.9) | 24 637 (45.4) |
| Multiparous | 194 (52.4) | 37 302 (55.7) | 63 (67.0) | 33 528 (55.2) | 43 (58.1) | 29 689 (54.6) |
| Marital status |  |  |  |  |  |  |
| Married/cohabitant | 332 (89.7) | 64 854 (96.9) | 91 (96.8) | 58 996 (97.2) | 72 (97.3) | 52 886 (97.3) |
| Other | 38 (10.3) | 2056 (3.1) | 3 (3.2) | 1730 (2.8) | 2 (2.7) | 1440 (2.7) |
| Pre-pregnancy BMI |  |  |  |  |  |  |
| <18.5 | 21 (5.7) | 1968 (2.9) | 5 (5.3) | 1737 (2.9) | 2 (2.7) | 1521 (2.8) |
| 18.5-24.9 | 233 (63.0) | 43 220 (64.6) | 61 (64.9) | 39 288 (64.7) | 43 (58.1) | 35 305 (65.0) |
| 25.0-29.9 | 81 (21.9) | 14 969 (22.4) | 21 (22.3) | 13 610 (22.4) | 17 (23.0) | 12 190 (22.4) |
| ≥30 | 35 (9.4) | 6723 (10.1) | 7 (7.5) | 6091 (10.0) | 12 (16.2) | 5310 (9.8) |
| Alcohol^a^ |  |  |  |  |  |  |
| No | 344 (93.0) | 64 939 (97.1) | 90 (95.7) | 58 986 (97.1) | 71 (96.0) | 52 756 (97.1) |
| Yes | 26 (7.0) | 1971 (2.9) | 4 (4.3) | 1740 (2.9) | 3 (4.0) | 1570 (2.9) |
| Smoking^b^ |  |  |  |  |  |  |
| No | 279 (75.4) | 59 776 (89.3) | 84 (89.4) | 54 534 (89.8) | 66 (89.2) | 49 018 (90.2) |
| Yes | 91 (24.6) | 7134 (10.7) | 10 (10.6) | 6192 (10.2) | 8 (10.8) | 5308 (9.8) |
| Educational level^c^ |  |  |  |  |  |  |
| 10-year primary school or less | 63 (17.0) | 5274 (7.9) | 10 (10.6) | 4413 (7.3) | 3 (4.0) | 3557 (6.6) |
| Secondary/vocational school | 100 (27.0) | 19 068 (28.5) | 23 (24.5) | 17 213 (28.3) | 25 (33.8) | 15 127 (27.8) |
| College or advanced degree | 207 (56.0) | 42 568 (63.6) | 61 (64.9) | 39 100 (64.4) | 46 (62.2) | 35 642 (65.6) |
| Family income, ITNR^d^ |  |  |  |  |  |  |
| <2 | 156 (42.2) | 23 846 (35.6) | 26 (27.7) | 21 324 (35.1) | 22 (29.7) | 18 714 (34.5) |
| ≥2 | 214 (57.8) | 43 064 (64.4) | 68 (72.3) | 39 402 (64.9) | 52 (70.3) | 35 612 (65.5) |
| Anxiety pre-pregnancy | 122 (33.0) | 2203 (3.3) | 21 (22.3) | 1906 (3.1) | 14 (18.9) | 1611 (3.0) |
| Depression pre-pregnancy | 141 (38.1) | 4196 (6.3) | 24 (25.5) | 3662 (6.0) | 17 (23.0) | 3129 (5.8) |
| Sleeping problems in early pregnancy | 197 (53.2) | 10 737 (16.0) | 25 (26.6) | 9560 (15.7) | 21 (28.4) | 8391 (15.4) |
| LTH of MD | 84 (22.7) | 4075 (6.1) | 16 (17.0) | 3549 (5.8) | 14 (18.9) | 3068 (5.6) |
| Chronic disease pre-pregnancy^e^ | 102 (27.6) | 8965 (13.4) | 27 (28.7) | 8042 (13.2) | 12 (16.2) | 7113 (13.1) |
| Benzodiazepine and/or z-hypnotic use during the 6 months prior to pregnancy | 164 (44.3) | 358 (0.5) | 10 (10.6) | 307 (0.5) | 5 (6.8) | 265 (0.5) |
| Symptoms of depression/anxiety at gestational week 17^f^, mean (SD) | - | - | 0.6 (1.2) | 0 (0.9) | 0.6 (1.4) | -0.1 (0.9) |
| Symptoms of depression/anxiety at gestational week 30^f^, mean (SD) | - | - | - | - | 0.9 (1.8) | -0.1 (0.9) |
| Comedication use during the 6 months prior to pregnancy |  |  |  |  |  |  |
| NSAIDs | 56 (15.1) | 6294 (9.4) | 12 (12.8) | 5797 (9.5) | 12 (16.2) | 5237 (9.6) |
| Opioids | 23 (6.2) | 972 (1.5) | 6 (6.4) | 865 (1.4) | 2 (2.7) | 765 (1.4) |
| Paracetamol | 114 (30.8) | 16 961 (25.3) | 35 (37.2) | 15 511 (25.5) | 24 (32.4) | 13 966 (25.7) |
| Antidepressants | 69 (18.6) | 909 (1.4) | 10 (10.6) | 793 (1.3) | 6 (8.1) | 678 (1.2) |
| Antipsychotics | 15 (4.1) | 104 (0.2) | - | 92 (0.2) | 1 (1.3) | 83 (0.2) |
| Antiepileptics^g^ | 8 (2.2) | 213 (0.3) | - | 184 (0.3) |  | 150 (0.3) |
| Comedication use in early pregnancy |  |  |  |  |  |  |
| NSAIDs | - | - | 6 (6.4) | 3065 (5.0) | 2 (2.7) | 2771 (5.1) |
| Opioids | - | - | 10 (10.6) | 743 (1.2) | 3 (4.0) | 648 (1.2) |
| Paracetamol | - | - | 60 (63.8) | 22 703 (37.4) | 37 (50.0) | 20 291 (37.4) |
| Antidepressants | - | - | 12 (12.8) | 550 (0.9) | 2 (2.7) | 467 (0.9) |
| Antipsychotics | - | - | 3 (3.2) | 417 (0.7) | 1 (1.3) | 370 (0.7) |
| Antiepileptics^g^ | - | - | - | 191 (0.3) |  | 157 (0.3) |
| Comedication use in mid pregnancy |  |  |  |  |  |  |
| NSAIDs | - | - | - | - | - | 585 (1.1) |
| Opioids | - | - | - | - | 4 (5.4) | 389 (0.7) |
| Paracetamol | - | - | - | - | 25 (33.8) | 12 427 (22.9) |
| Antidepressants | - | - | - | - | 3 (4.0) | 177 (0.3) |
| Antipsychotics | - | - | - | - | - | 110 (0.2) |
| Antiepileptics^g^ | - | - | - | - | - | 57 (0.1) |

Abbreviations: BMI, body mass index; ITNR, income to needs ratio; LTH of MD, Life Time History of Major Depression; NSAIDs, nonsteroidal anti-inflammatory drugs.

^a^ Alcohol use was measured in MoBa questionnaire 1.

^b^ Smoking was measured in early pregnancy.

^c^ Educational level assessed in the child’s birth year.

^d^ Family income was assessed by ITNR

^e^ Chronic disease included asthma, diabetes, hypertension, epilepsy, arthritis, other heart disease, thyroid disorder, lupus or Crohn’s disease, reported before pregnancy.

^f^ Presence of symptoms of depression or anxiety indicated on the 5-item short version of the Hopkins Symptoms Checklist (SCL-5)

^g^ Antiepileptic use does not include clonazepam (N03AE01).

**Table S15. Results from sensitivity analysis, changing the eligibility criteria to include all MoBa participants without requiring a history of anxiety and/or depression.** Crude and weighted mean differences in the children’s test scores in literacy and numeracy according to the mothers’ initiation of benzodiazepines or z-hypnotics in each trial (early pregnancy trial N=67 280, mid pregnancy trial N =60 820, late pregnancy trial N=54 400)

|  | **N** | **Mean z score (SD)** | **Crude mean difference (95% CI)** | **Weighted mean difference (95% CI)** |
| --- | --- | --- | --- | --- |
| **Literacy** |  |  |  |  |
| Treatment initiation in the **early** pregnancy trial | 370 | 0.19 (0.9) | -0.01 (-0.11, 0.09) | 0.01 (-0.21, 0.22) |
| No treatment initiation in the **early** pregnancy trial | 66 910 | 0.20 (1.0) |  |  |
| Treatment initiation in the **mid** pregnancy trial | 94 | 0.07 (1.0) | -0.14 (-0.37, 0.08) | -0.05 (-0.45, 0.34) |
| No treatment initiation in the **mid** pregnancy trial | 60 726 | 0.21 (1.0) |  |  |
| Treatment initiation in the **late** pregnancy trial | 74 | 0.10 (1.0) | -0.13 (-0.37, 0.10) | -0.16 (-0.44, -0.15) |
| No treatment initiation in the **late** pregnancy trial | 54 326 | 0.23 (1.0) |  |  |
| **Numeracy** |  |  |  |  |
| Treatment initiation in the **early** pregnancy trial | 370 | 0.10 (1.0) | -0.11 (-0.21, -0.01) | -0.02 (-0.23, 0.19) |
| No treatment initiation in the **early** pregnancy trial | 66 910 | 0.21 (1.0) |  |  |
| Treatment initiation in the **mid** pregnancy trial | 94 | 0.11 (0.9) | -0.11 (-0.30, 0.08) | -0.04 (-0.32, 0.28) |
| No treatment initiation in the **mid** pregnancy trial | 60 726 | 0.22 (1.0) |  |  |
| Treatment initiation in the **late** pregnancy trial | 74 | 0.25 (1.0) | 0.01 (-0.22, 0.26) | 0.02 (-0.42, 0.40) |
| No treatment initiation in the **late** pregnancy trial | 54 326 | 0.24 (1.0) |  |  |

**Table S16. Results from sensitivity analysis, for new cases of anxiety and/or depression in early or mid pregnancy.** Crude and weighted mean differences in the children’s test scores in literacy and numeracy according to the mothers’ initiation of benzodiazepines or z-hypnotics in the mid and late pregnancy trial.

|  | **N** | **Mean z score (SD)** | **Crude mean difference (95% CI)** | **Weighted mean difference (95% CI)** |
| --- | --- | --- | --- | --- |
| **Literacy** |  |  |  |  |
| Treatment initiation in the **mid** pregnancy trial | 30 | -0.04 | -0.14 (-0.53, 0.23) | -0.10 (-1.02, 0.90) |
| No treatment initiation in the **mid** pregnancy trial | 4545 | 0.10 |  |  |
| Treatment initiation in the **late** pregnancy trial | 11 | 0.15 | 0.04 (-0.51, 0.28) | 0.15 (-0.71, 0.74) |
| No treatment initiation in the **late** pregnancy trial | 1877 | 0.11 |  |  |
| **Numeracy** |  |  |  |  |
| Treatment initiation in the **mid** pregnancy trial | 30 | -0.10 | -0.15 (-0.43, 0.14) | -0.19 (-0.72, 0.85) |
| No treatment initiation in the **mid** pregnancy trial | 4545 | 0.06 |  |  |
| Treatment initiation in the **late** pregnancy trial | 11 | 0.24 | 0.19 (-0.41, 0.50) | -0.10 (-0.76, 1.76) |
| No treatment initiation in the **late** pregnancy trial | 1877 | 0.05 |  |  |

**Table S17. Results from sensitivity analysis: Continuation vs. discontinuation in early pregnancy among women who used benzodiazepines or z-hypnotics during the 6 months prior to pregnancy.** Crude and weighted mean differences in the children’s test scores in literacy and numeracy according to the mothers’ continuation or discontinuation of benzodiazepines or z-hypnotics in early pregnancy.

|  | **N** | **Mean z score (SD)** | **Crude mean difference (95% CI)** | **Weighted mean difference (95% CI)** |
| --- | --- | --- | --- | --- |
| **Literacy** |  |  |  |  |
| Continuation in early pregnancy | 155 | 0.20 | -0.08 (-0.27, 0.10) | -0.04 (-0.24, 0.15) |
| Discontinuation before early pregnancy | 351 | 0.28 |  |  |
| **Numeracy** |  |  |  |  |
| Continuation in early pregnancy | 155 | 0.06 | -0.20 (-0.39, -0.01) | -0.17 (-0.39, 0.03) |
| Discontinuation before early pregnancy | 351 | 0.26 |  |  |

|  | **N** | **Mean z score (SD)** | **Crude mean difference (95% CI)** | **Weighted mean difference (95% CI)** |
| --- | --- | --- | --- | --- |
| **Literacy** |  |  |  |  |
| Continuation in early pregnancy | 97 | 0.30 | 0.03 (-0.21, 0.26) | 0.04 (-0.23, 0.32) |
| Discontinuation before early pregnancy | 210 | 0.27 |  |  |
| **Numeracy** |  |  |  |  |
| Continuation in early pregnancy | 97 | 0.04 | -0.22 (-0.47, 0.03) | -0.25 (-0.57, 0.05) |
| Discontinuation before early pregnancy | 210 | 0.26 |  |  |

Table S18. Results from sensitivity analysis: Continuation vs. discontinuation in early pregnancy among women who used benzodiazepines or z-hypnotics during the 6 months prior to pregnancy and have a history of anxiety and/or depression prior to pregnancy. Crude and weighted mean differences in the children’s test scores in literacy and numeracy according to the mothers’ continuation or discontinuation of benzodiazepines or z-hypnotics in early pregnancy.

**Table S19. Results from sensitivity analyses, pooling the trials in the main analysis.** Crude and weighted mean differences in the children’s test scores in literacy and numeracy according to the mothers’ initiation of benzodiazepines or z-hypnotics in pregnancy.

|  | **N** | **Mean z score (SD)** | **Crude mean difference (95% CI)** | **Weighted mean difference (95% CI)** |
| --- | --- | --- | --- | --- |
| **Literacy** |  |  |  |  |
| Treatment initiation | 242 | 0.14 | -0.07 (-0.20, 0.05) | -0.06 (-0.24, 0.10) |
| No treatment initiation | 7553 | 0.21 |  |  |
| **Numeracy** |  |  |  |  |
| Treatment initiation | 242 | 0.03 | -0.12 (-0.26, 0.02) | -0.13 (-0.35, 0.10) |
| No treatment initation | 7553 | 0.15 |  |  |


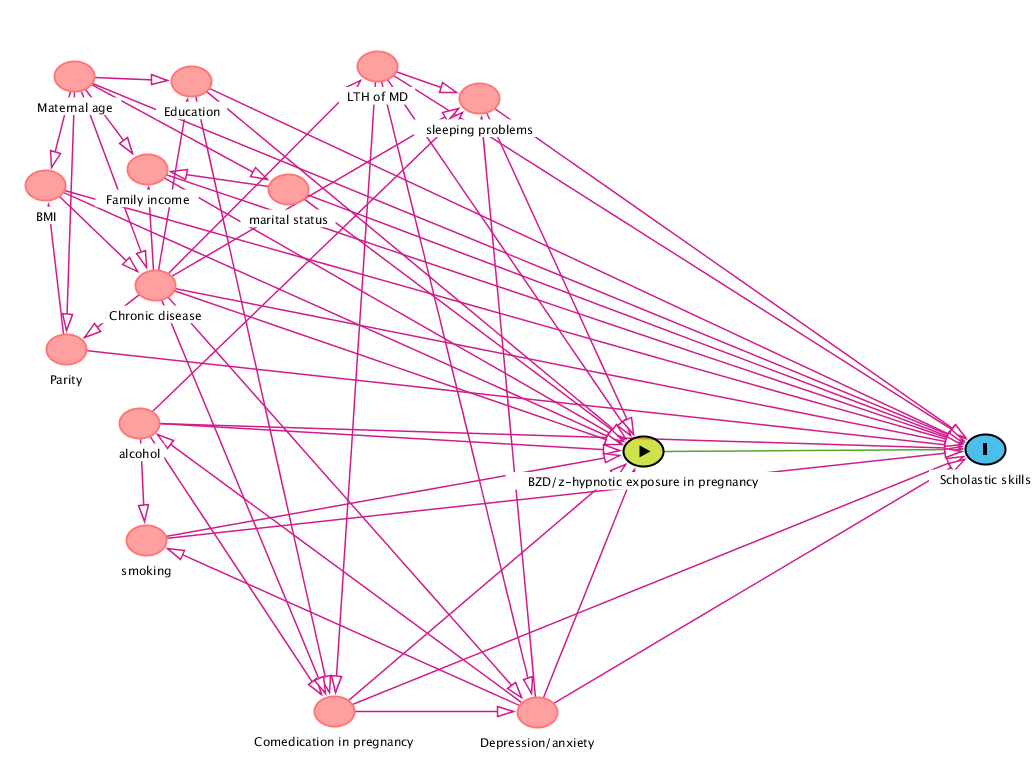


**Figure S1. Directed acyclic graph showing assumed covariate structure, drawn as a time-fixed model for simplicity.**

Abbreviations: BMI, body mass index; BZD, benzodiazepine; LTH of MD, Life Time History of Major Depression.


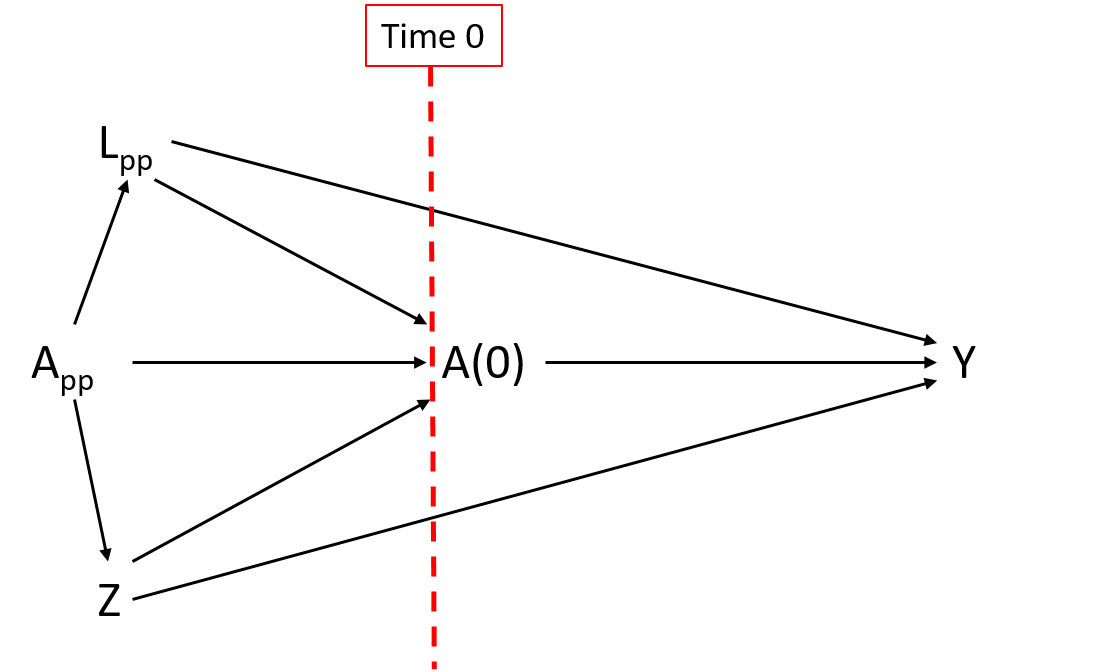


Figure S2. DAG for the early pregnancy trial.

Time 0 corresponds to the time the treatment is measured, eligibility is assessed, and follow up begins, i.e. gestational week 0. PP corresponds to the period from 6 months prior to pregnancy to gestational week 0. A = treatment with benzodiazepines and/or z-hypnotics, L = time-varying covariates, Z = baseline covariates, Y = outcome.


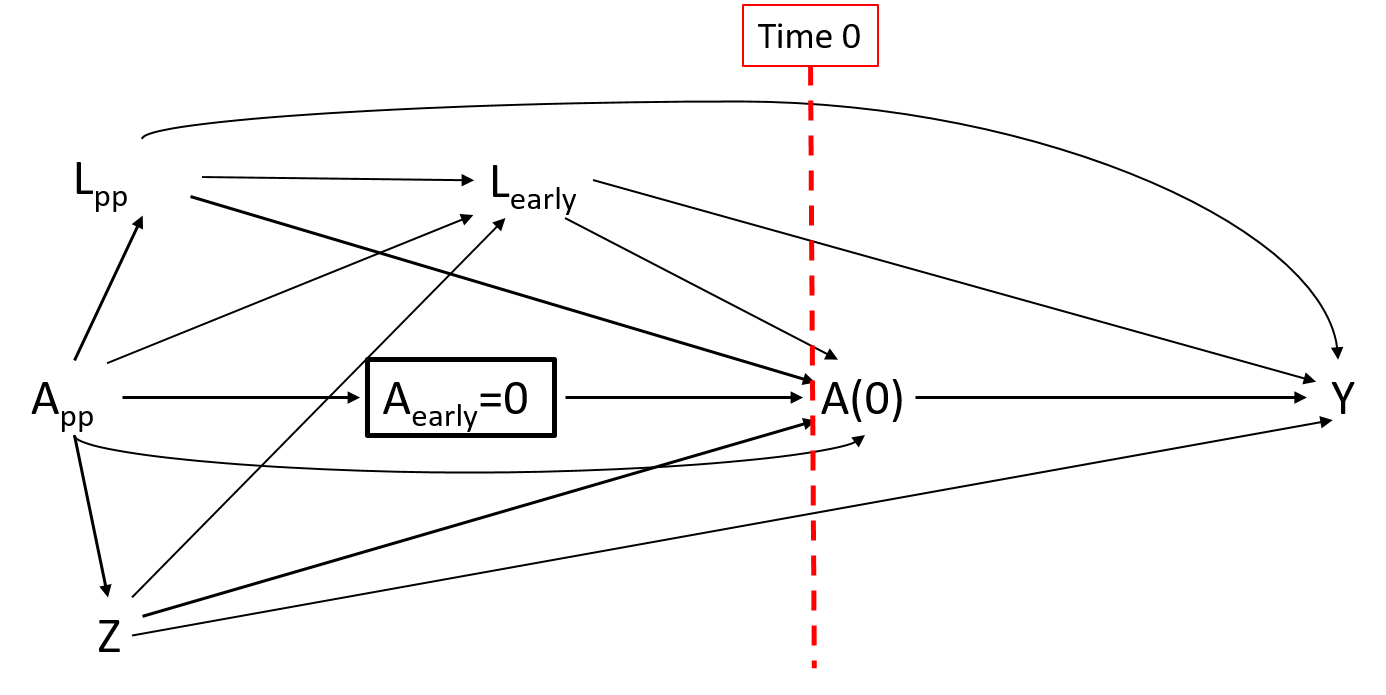


Figure S3. DAG for the mid pregnancy trial.

Time 0 corresponds to the time the treatment is measured, eligibility is assessed, and follow up begins, i.e. gestational week 17. PP corresponds to the period from 6 months prior to pregnancy to gestational week 0; Early corresponds to the period from gestational week 0 to 16. A = treatment with benzodiazepines and/or z-hypnotics, L = time-varying covariates, Z = baseline covariates, Y = outcome.


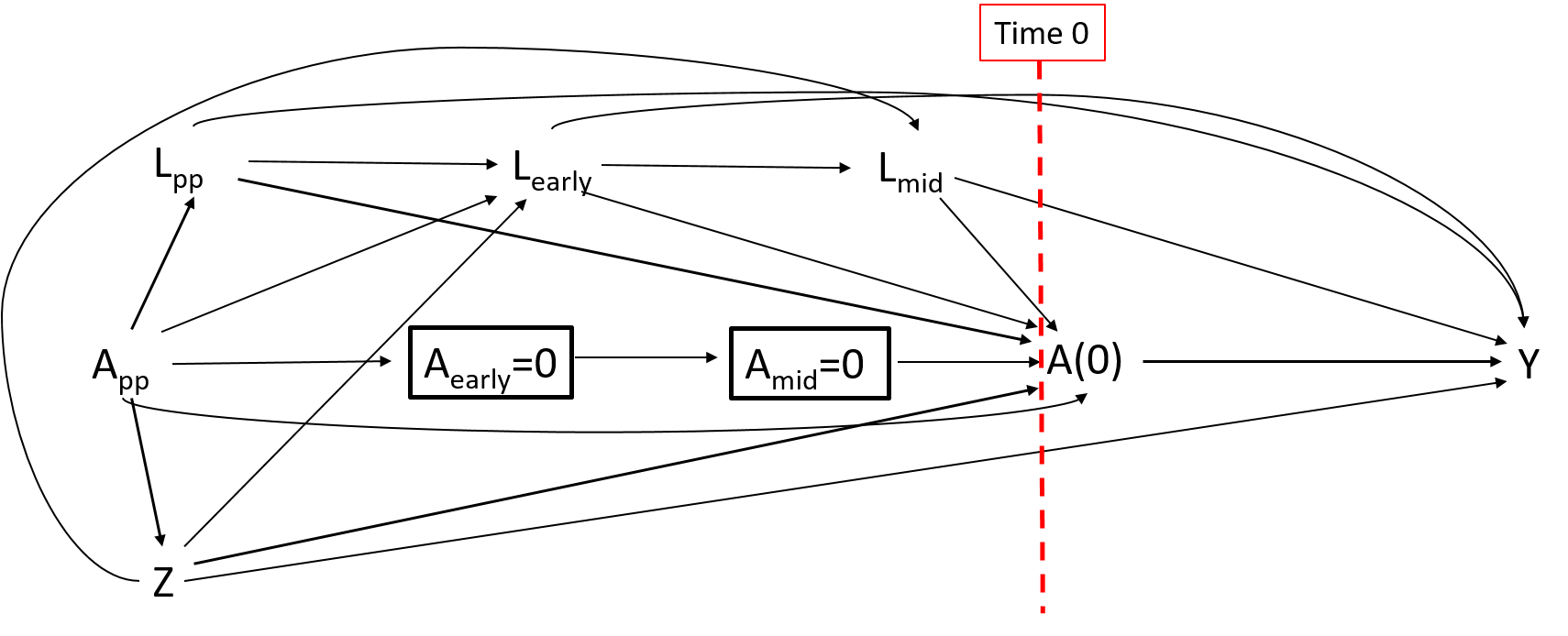


Figure S4. DAG for the late pregnancy trial.

Time 0 corresponds to the time the treatment is measured, eligibility is assessed, and follow up begins, i.e. gestational week 29. PP corresponds to the period from 6 months prior to pregnancy to gestational week 0; Early corresponds to the period from gestational week 0 to 16; Mid corresponds to the period from gestational week 17 to 28. A = treatment with benzodiazepines and/or z-hypnotics, L = time-varying covariates, Z = baseline covariates, Y = outcome.


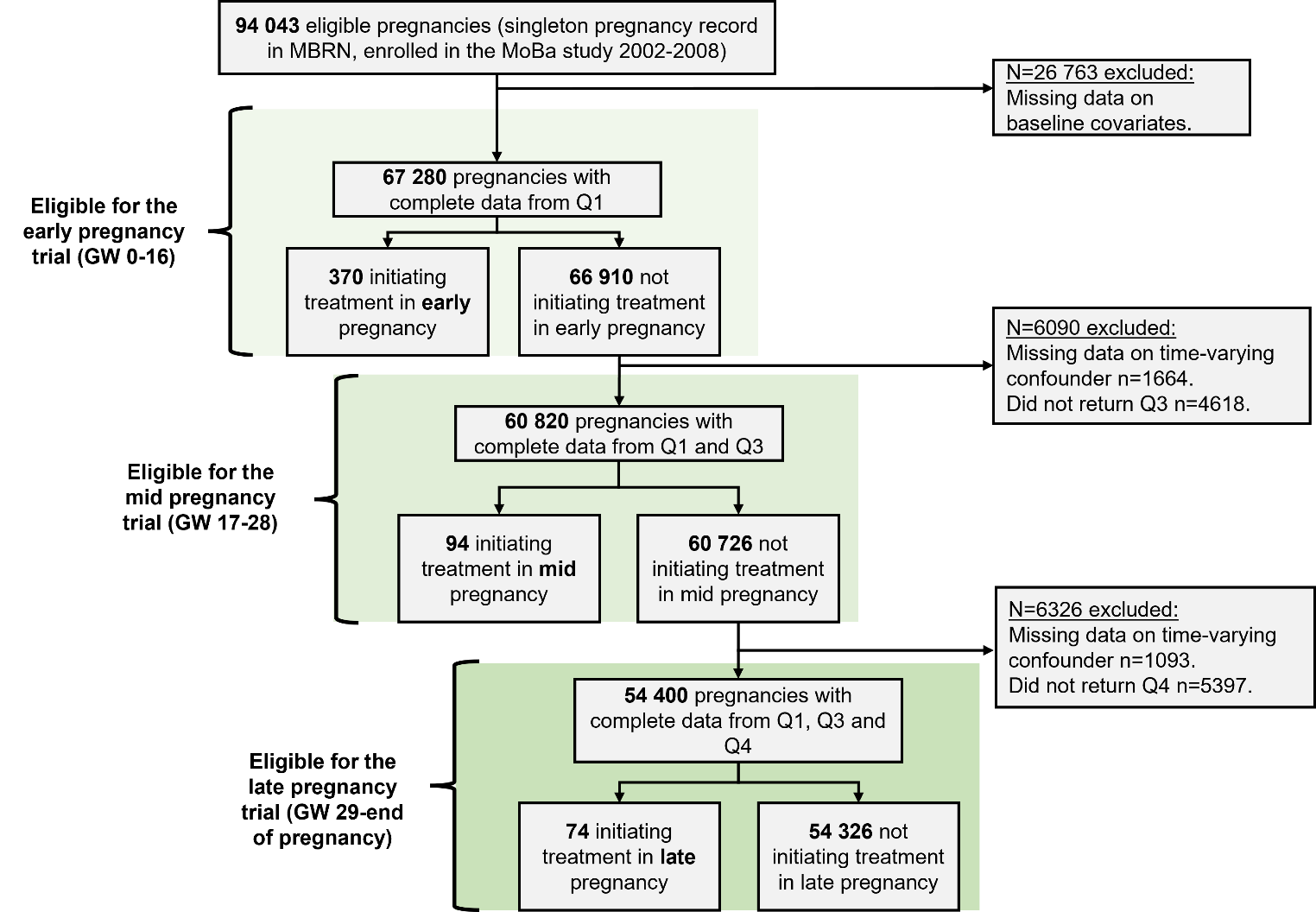


Figure S5. Flowchart showing the inclusion and exclusion criteria for the emulated early, mid and late pregnancy trials in the sensitivity analysis, changing the eligibility criteria to include all MoBa participants without requiring a history of anxiety or depression.

MoBa children born between 1999-2001 were not included in this study because of lack of consent (they became 18 years before the follow-up was completed). Conditions of exclusion can overlap. Abbreviations: GW, gestational week; MBRN, Medical Birth Registry of Norway; MoBa, Norwegian Mother, Father and Child Cohort Study; Q1, MoBa questionnaire 1; Q3, MoBa questionnaire 3; Q4, MoBa questionnaire 4.


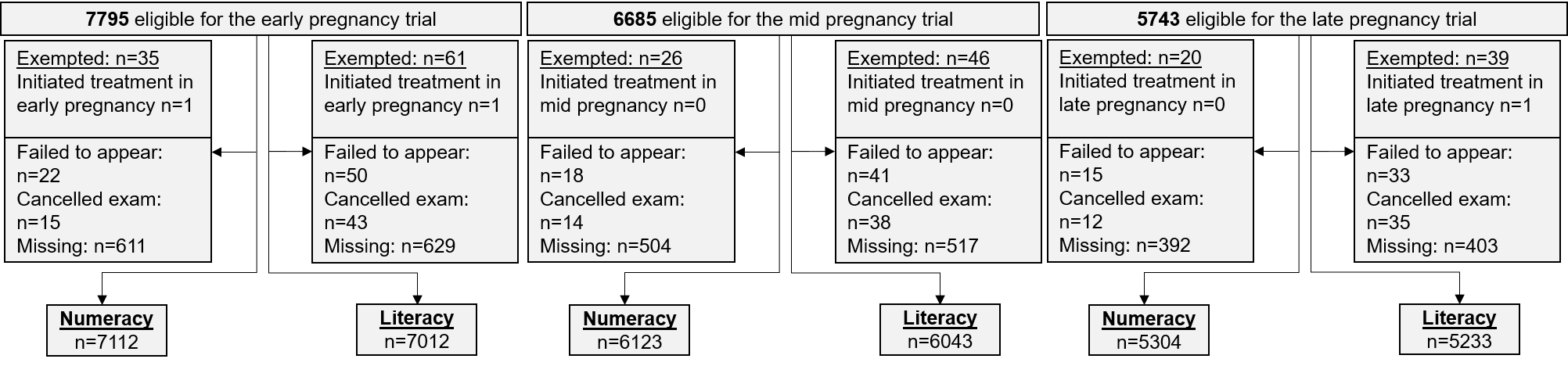


Figure S6. Overview of the number of exempted children in each subject, in each trial.


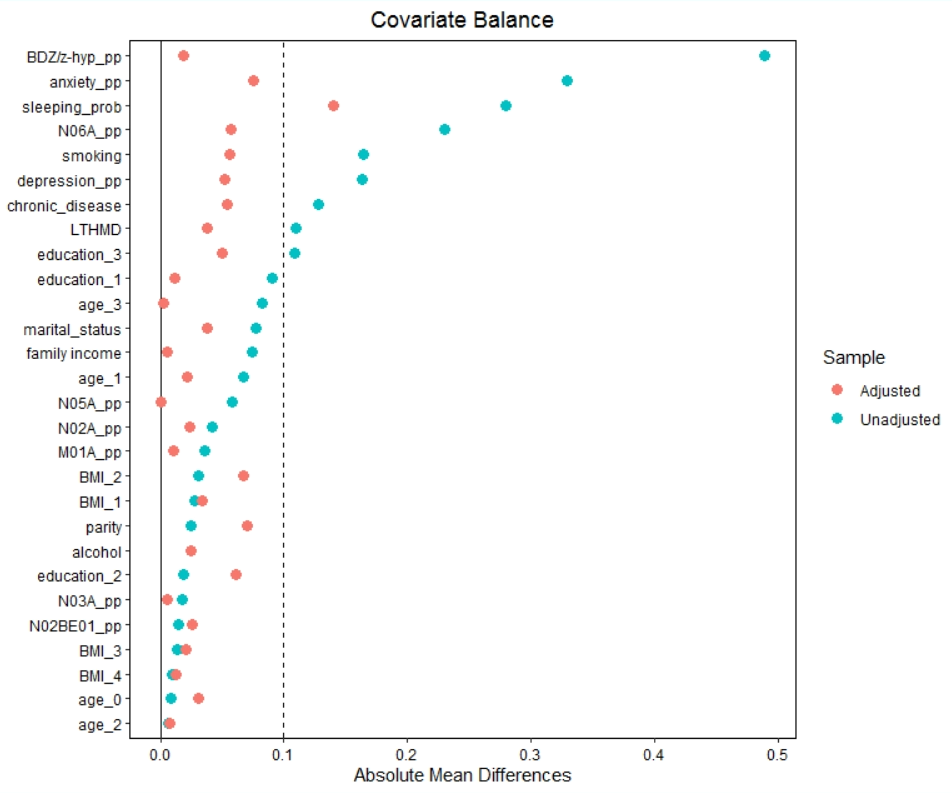


Figure S7. Balance before and after IPTW of covariates between individuals who initiated treatment vs. not initiated treatment in the early pregnancy trial. Sleeping problems remained imbalanced after weighting, and was included in the outcome models.

Abbreviations: BMI, body mass index; BZD, benzodiazepine; IPTW, inverse probability of treatment weighting; LTH of MD, Life Time History of Major Depression; pp, pre-pregnancy; z-hyp, z-hypnotics.

**
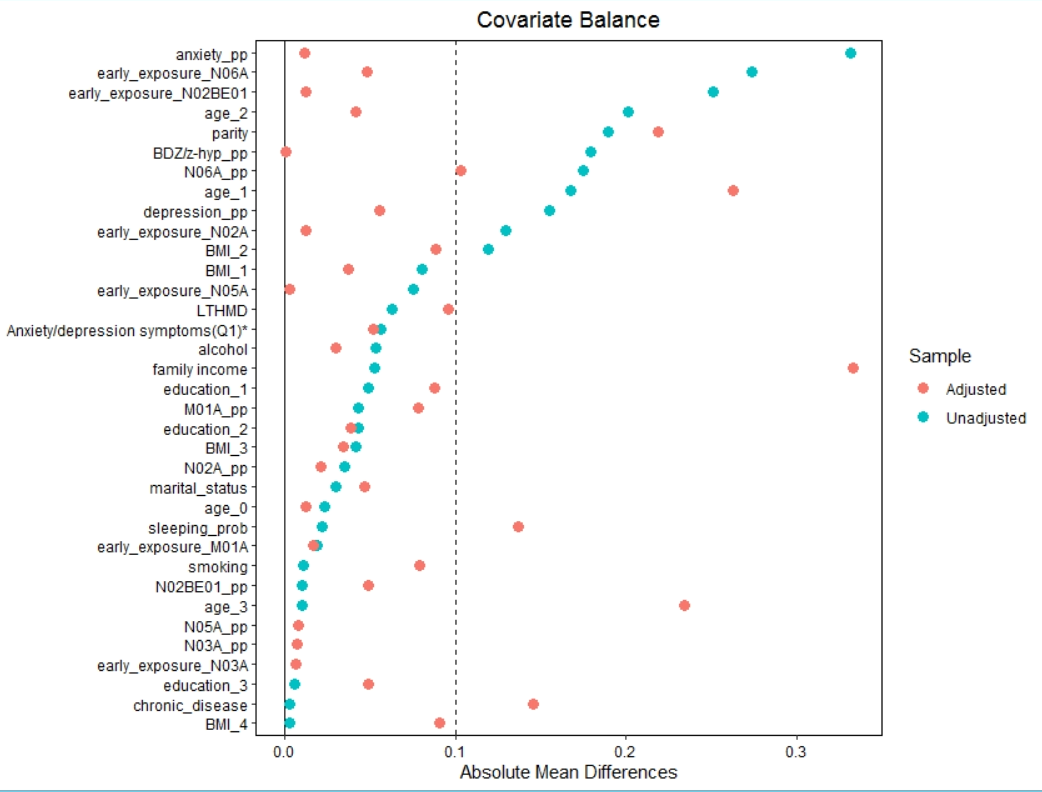
**

Figure S8. Balance before and after IPTW of covariates between individuals who initiated treatment vs. not initiated treatment in the mid pregnancy trial. Parity, maternal age, family income, sleeping problems, and chronic disease remained imbalanced after weighting, and were included in the outcome models.

Abbreviations: BMI, body mass index; BZD, benzodiazepine; IPTW, inverse probability of treatment weighting; LTH of MD, Life Time History of Major Depression; pp, pre-pregnancy; Q1, MoBa questionnaire 1; z-hyp, z-hypnotics.


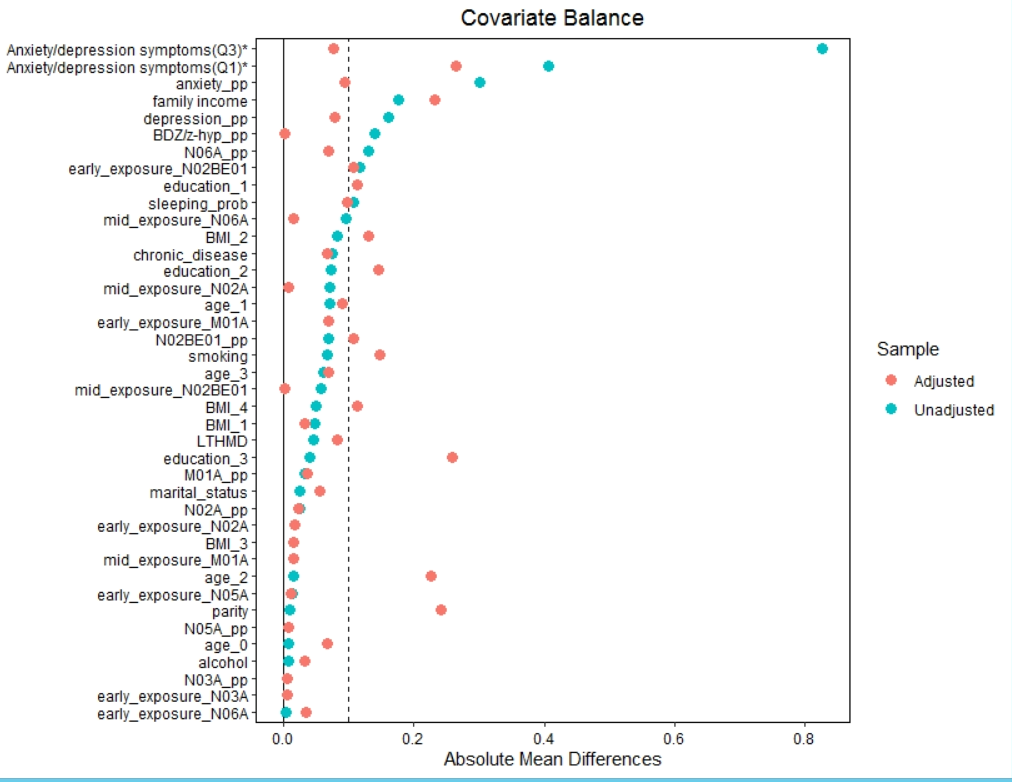


Figure S9. Balance before and after IPTW of covariates between individuals who initiated treatment vs. not initiated treatment in the late pregnancy trial. Symptoms of anxiety and depression (Q1), family income, education, parity, smoking and maternal age remained imbalanced after weighting, and were included in the outcome models.

Abbreviations: BMI, body mass index; BZD, benzodiazepine; IPTW, inverse probability of treatment weighting; LTH of MD, Life Time History of Major Depression; pp, pre-pregnancy; Q1, MoBa questionnaire 1; Q3, MoBa questionnaire 3; z-hyp, z-hypnotics.
